# Anxiolytic effect of GABAergic neurons in the anterior cingulate cortex in a rat model of chronic inflammatory pain

**DOI:** 10.1186/s13041-021-00849-9

**Published:** 2021-09-10

**Authors:** Fang-bing Shao, Jun-fan Fang, Si-si Wang, Meng-ting Qiu, Dan-ning Xi, Xiao-ming Jin, Jing-gen Liu, Xiao-mei Shao, Zui Shen, Yi Liang, Jian-qiao Fang, Jun-ying Du

**Affiliations:** 1 Department of Neurobiology and Acupuncture Research, the Third School of Clinical Medicine, Zhejiang Chinese Medical University, Key Laboratory of Acupuncture and Neurology of Zhejiang Province, Hangzhou, 310053 China; 2 Department of Anatomy and Cell Biology, Stark Neurosciences Research Institute, Indiana University School of Medicine, NB Building, 320w 15th Street #141, Indianapolis, IN 46202 USA; 3 Key Laboratory of Receptor Research, Shanghai Institute of Materia Medica, Chinese Academy of Sciences, Shanghai, 201203 China

**Keywords:** Chronic inflammatory pain, Anxiety-like behavior, GABAergic system, Anterior cingulate cortex; synaptic transmission

## Abstract

Chronic pain easily leads to concomitant mood disorders, and the excitability of anterior cingulate cortex (ACC) pyramidal neurons (PNs) is involved in chronic pain-related anxiety. However, the mechanism by which PNs regulate pain-related anxiety is still unknown. The GABAergic system plays an important role in modulating neuronal activity. In this paper, we aimed to study how the GABAergic system participates in regulating the excitability of ACC PNs, consequently affecting chronic inflammatory pain-related anxiety. A rat model of CFA-induced chronic inflammatory pain displayed anxiety-like behaviors, increased the excitability of ACC PNs, and reduced inhibitory presynaptic transmission; however, the number of GAD65/67 was not altered. Interestingly, intra-ACC injection of the GABA_A_R agonist muscimol relieved anxiety-like behaviors but had no effect on chronic inflammatory pain. Intra-ACC injection of the GABA_A_R antagonist picrotoxin induced anxiety-like behaviors but had no effect on pain in normal rats. Notably, chemogenetic activation of GABAergic neurons in the ACC alleviated chronic inflammatory pain and pain-induced anxiety-like behaviors, enhanced inhibitory presynaptic transmission, and reduced the excitability of ACC PNs. Chemogenetic inhibition of GABAergic neurons in the ACC led to pain-induced anxiety-like behaviors, reduced inhibitory presynaptic transmission, and enhanced the excitability of ACC PNs but had no effect on pain in normal rats. We demonstrate that the GABAergic system mediates a reduction in inhibitory presynaptic transmission in the ACC, which leads to enhanced excitability of pyramidal neurons in the ACC and is associated with chronic inflammatory pain-related anxiety.

**Supplementary Information:**

The online version contains supplementary material available at 10.1186/s13041-021-00849-9.

## Introduction

Chronic inflammatory pain is one of the major problems that affects the quality of life of patients. Scientists have developed diverse research and have made some progresses in understanding its pathological mechanism and intervention [1–4]. However, the treatment of chronic inflammatory pain is still a considerable challenge in clinical practice. Furthermore, chronic pain easily leads to concomitant mood disorders, such as anxiety and depression, and morbidity ranges from 20 to 40% in chronic pain patients [5, 6], which make the mechanism underlying chronic inflammatory pain much more complex. In addition, negative emotion threatens the quality of life of chronic pain patients far more than chronic pain does in some cases. Therefore, it is particularly important to explore the mechanism of chronic inflammatory pain-related negative emotion to provide patients with better treatment options.

It has been proposed that the anterior cingulate cortex (ACC) is an important central hub for pain and pain-related emotion [7, 8]. A growing number of studies have shown that the ACC displays a series of structural and functional changes under chronic pain [9], and neurons in the ACC can be activated by nociceptive stimuli [10]. In addition, inhibition of excitatory neurons in the ACC by photogenetic techniques could produce antianxiety effects associated with chronic pain [11]. All these results indicate that ACC neuron activation tightly links chronic inflammatory pain and negative emotions. However, the neuronal substrates underlying the activation of ACC neurons are still elusive.

Normal neuronal functions rely on a delicate balance between excitation and inhibition. As a major inhibitory neurotransmitter in the nervous system, γ-aminobutyric acid (GABA) plays an important role in modulating neuronal activity [12]. Previous studies suggested that insufficient GABA synthesis in several brain regions induces the hyperactivation of neurons and produces anxiety and other negative emotional reactions [13]. In addition, reduced GABAergic transmission in the ACC is involved in chronic inflammatory and neuropathic pain [9, 14, 15]. Furthermore, dysfunction of type A γ-aminobutyric acid receptor (GABA_A_R)-mediated inhibitory function in the ACC is involved in the affective of the pain experience, and GABAergic cell transplantation into the ACC reduces neuropathic pain aversiveness [16]. A previous study demonstrated that presynaptic spontaneous GABAergic plasticity of the ACC was changed in the induction phase of chronic inflammatory pain [17]. However, it is not fully understood whether GABAergic transmission is changed in the persistent phase of chronic pain and whether it is involved in chronic inflammatory pain-related anxiety.

In this study, we aimed to determine how the GABAergic system participates in regulating the excitability of ACC pyramidal neurons (PNs) to affect chronic inflammatory pain-related anxiety. For this purpose, we established a chronic inflammatory pain model by complete Freund’s adjuvant (CFA) injection, then observed pain behavior and anxiety-like behaviors. Molecular, pharmacological, electrophysiological, and chemogenetic methods were combined to determine the mechanism by which the GABAergic system modulates the excitability of ACC PNs in pain-related anxiety. Furthermore, we investigated the average frequency and amplitude of spontaneous inhibitory synaptic currents (sIPSCs) by acute slice patch-clamp recording combined with chemogenetic methods to confirm the connection between GABA release and the excitability of ACC PNs.

## Methods

### Animals

Healthy adult male Sprague–Dawley (SD) rats weighing 200 g to 220 g were provided by the Shanghai Laboratory Animal Center, Chinese Academy of Sciences. A maximum of four rats were housed per cage with free access to food and water. The rats kept in a suitable environment (temperature: 25 ± 2 ℃, humidity: 40–60%) with a 12 h light/dark cycle. The rats were allowed to adapt to their housing environment for one week before any experiments. All experimental protocols received approval from the Animal Care and Welfare Committee of Zhejiang Chinese Medical University, Zhejiang, China (IACUC-20180319-12).

### Chronic inflammatory pain model

Except for the control rats, the chronic inflammatory pain model was established in rats by subcutaneously injecting 0.1 mL complete Freund’s adjuvant (CFA, Sigma, USA) into the left hind paw of the rats. The control rats were injected with the same amount of saline at the same site.

### Behavioral assessment of mechanical hyperalgesia and anxiety

#### Paw withdrawal thresholds (PWTs)

The PWTs were measured using von Frey hairs (North Coast, USA) at multiple time points (base, 1 days, 7 days, 14 days, 21 days and 28 days after CFA injection), the procedure for which was described by Chaplan et al. [6]. Rats were placed in a transparent plastic box (20 cm × 20 cm × 15 cm) on an elevated mesh floor and allowed to adapt to the test environment for 30 min in a quiet environment. Von Frey hairs were applied in a series of ascending forces (0.4, 0.6, 1, 2, 4, 6, 8, 15, and 26 g) to the central surface of the hindpaw, with sufficient force to bend the hair slightly for 6–8 s. The first hair applied corresponded to a force of 4 g. When the hindpaw of the rats was stimulated, abrupt withdrawal or licking or shaking of the foot was considered a pain-like response and the result was marked as “X”. Then, a weaker stimulus was chosen. If there was no response, the result was marked as “O” and the force was changed to the next strongest force. After the first “OX or XO” combination appeared, we still needed to record four datapoints. The PWTs were calculated using the following formula: PWTs (g) = 10 (xf + k * δ − 4). If PWTs > 26 g or < 0.4 g were calculated, 26 g or 0.4 g was still taken as the maximum or minimum value. “Xf” was the value of the von Frey hair last used, “k” was the corresponding value of the resulting sequence in the k-value table, and “δ” was the mean difference between stimuli (here, 0.231). The interval between each stimulus was not less than 2 min in all rats.

#### Open field test (OF)

The experiment was conducted in a square box (100 cm × 100 cm × 50 cm) without a lid at 29 d after CFA injection. At the beginning of the test, rats were individually placed in the middle of a box with dim light and were allowed to freely orient themselves to the arena for 5 min while being recorded. Smart 3.0 software was used to analyze the video. The data collected included the total distance traveled, percentage of distance traveled in the central area, time in the central area and the number of central area entries. To eliminate the olfactory stimulus of animals’ odor between sessions, the test box was washed with 10% alcohol before each measurement to remove the olfactory stimuli such as odor and stool left by the previous rat.

#### Elevated zero maze test (EZM)

The test was measured at 30 d after CFA injection on a ring-shaped apparatus (100 cm × 50 cm × 25 cm), consisting of two open arms and two closed arms that had a dim light. Each rat was placed between the open arm and closed arm, facing the open arm; the rat was allowed to investigate the maze for 5 min and was recorded. The collected data included the percentage of distance traveled in the open arm, time spent in the open arm and the number of open arm entries. Smart 3.0 software was used to analyze the video. To eliminate the olfactory stimulus of animals’ odor between sessions, the test box was washed with 10% alcohol before each measurement to eliminate the olfactory stimuli, such as odor and stool, left by the previous rat.

#### Novelty suppressed feeding test (NSF)

The test was measured at 31 d after CFA injection, consisted of a black homemade box (40 cm × 40 cm × 30 cm) with 1 cm thick bedding at the bottom. All rats were food restricted but not water restricted for 24 h. During the test, the environment needed to be bright, and a food pellet was placed on a piece of paper at the center of the box. The rats were placed separately in any corner, and the time when the rats first picked up and ate food was recorded. The latency to feed was recorded with a maximum time of 5 min. After the rat began to eat food, the rat was immediately transferred to its cage. Then, a five-minute food consumption test was conducted to rule out the effect of differences in appetite on feed latency.

#### Marble-burying test (MBT)

The test was performed in a cage (20 cm × 40 cm × 25 cm) covered with 5 cm thick bedding at 32 d after CFA injection. The cage was equipped with 9 marbles with a diameter of 2.5 cm, which were arranged in a of “3 × 3 × 3” pattern. The rats were individually placed at any corner in a cage, and the number of marbles buried by the rat within 10 min was recorded. A marble was considered buried if 2/3 of it was covered with bedding.

### Drugs

Clozapine-N-oxide (CNO, 2 mg/kg, Wuhan Brain TVA, China) was dissolved in 0.5% dimethylsulfide (DMSO, Sigma, USA). The specific GABA_A_R agonist muscimol (Sigma, USA) and specific GABA_A_R antagonist picrotoxin (Sigma, USA) were freshly dissolved in artificial cerebrospinal fluid consisting of (in millimolar): NaCl 137, CaCl_2_ 1.2, KCl 3, MgSO_4_ 1, NaH_2_PO_4_ 0.5, Na_2_HPO_4_ 2, and glucose 3; its pH 7.3 and its final concentration was 1 μg/μl. Then, the ACC was injected with 0.5 μl of the drug volume. Doses of CNO, muscimol and picrotoxin were based on previous studies demonstrating their modulating effects in behavioral tasks [18–20].

### Adeno-associated virus (AAV)

To regulate the activity of GABAergic interneurons in the ACC, we bilaterally injected rAAV-VGAT1-hM4D(Gi)-mCherry-WPRE-pA (virus titers: 2.02E+12 vg/ml) in naïve rats and unilaterally injected rAAV-VGAT1-hM3D(Gq)-mCherry-WPRE-pA (virus titers: 3.27E+12 vg/ml) into the rACC in model rats. In the mCherry (null virus) control rats, the virus used was rAAV-VGAT1-mCherry-WPRE-pA (virus titers: 2.58E+12 vg/ml). All viruses we used in the study were purchased from Wuhan Brain TVA Co., Ltd.

### Stereotactic injection and intracranial injections

Rats were deeply anesthetized with 2% isoflurane and placed in a stereotactic frame (RWD) for a craniotomy. In the chemogenetic procedures, 400 nl AAV2/9 virus was injected into the ACC (AP: + 2.76 mm, ML: ± 0.75 mm, DV: + 1.4 mm) at a rate of 50 nl/min using a WPI nanofill syringe (10 μl) connected with a microsyringe pump controller (WPI, USA). After the injection, the needle was held still for 10 min to allow for diffusion then slowly pulled out; afterwards, the skin was sewn back together. All viruses were allowed to express for 4 weeks for maximum results.

For intracranial injections, rats were unilaterally implanted with guide cannulae (OD 0.48 mm, C = 6 mm, RWD) in the ACC (AP: + 2.76 mm, ML: ± 0.75 mm, DV: + 1.4 mm), the guide cannulae were fixed to the skull with four stainless-steel screws and dental cement. To prevent clogging and reduce the risk of infection, a catheter cap 0.5 mm longer than the catheter was inserted. Before drug delivery, the injector cannula (OD 0.30 mm, C = 6 mm, G = 0.5 mm, RWD) was inserted into the guide cannulae and connected to the WPI nanofill syringe (10 μl). The ACC was injected with 0.5 μl of the drug volume at a speed of 200 nl/min. After the injection, the needle remained in place for 3 min to allow for diffusion, and rats were placed in their cages for 20 min before they underwent the behavioral test. We confirmed cannula placement under a light microscope and verified the location of virus injection by the mCherry signal in the ACC of brain slices under a fluorescence microscope.

### Immunofluorescence (IF)

After the last behavioral test, rats were sacrificed within 2 h of CNO injection. The rats were deeply anesthetized with pentobarbital (80 mg/kg, i.p.) and perfused transcardially with 0.1 M PBS and 4% paraformaldehyde. The brains were removed and fixed in 4% paraformaldehyde for 24 h and then transferred to 15% and 30% sucrose solutions until they sank completely to ensure tissue dehydration. Brains were embedded in Tissue-Tek O.C.T (Thermo, USA) and cut into 30 μm tissue sections on a cryostat microtome NX50 (Thermo, USA) for IF. Sections were washed four times in TBST (10 min each) and then blocked with 0.3% Triton X-100 and 5% normal donkey serum for 1 h at 37 ℃. Then the sections were incubated with rabbit anti-c-Fos (1:500, ab190289, Abcam, USA), mouse anti-c-Fos (1:500, ab208942, Abcam, USA) rabbit anti-GAD65/67 (1:200, Abcam, ab183999, UA), and mouse anti-CaMKII (1:200, ab22609, Abcam, USA) antibodies overnight at 4 ℃. The next day, the sections were washed three times in TBST and incubated with preadsorbed secondary donkey anti-rabbit IgG H&L (Alexa Fluor 488) (1:500, ab150061, Abcam, USA), or preadsorbed donkey anti-rabbit IgG H&L (Alexa Fluor 488) (1:500, ab150061, Abcam, USA) or preadsorbed goat anti-mouse IgG H&L (Cy3) (1:500, ab97035, Abcam, USA) for 1 h at 37 ℃. Sections were washed three times in TBST, and images were acquired using an Imager M2 microscope (ZEISS, Germany). The number of positive cells in the ACC region was counted manually in 5 sections of each rat, and the boundaries of this anatomical region were determined through the rat brain atlas (The Rat Brain in Stereotaxic Coordinates, 6^th^ Edition Paxinos & Watson).

### Preparation of ACC slices

The rats were deeply anesthetized with pentobarbital (80 mg/kg, i.p.) and then quickly decapitated so that their brains could be removed. The brains were quickly placed in ice-cold oxygenated (95% O_2_/5% CO_2_) cutting solution containing (mM) 125 NaCl, 2.5 KCl, 0.1 CaCl_2_, 3.9 MgCl_2_, 26 NaHCO_3_, 1.25 NaH_2_PO_4_-H_2_O, 2.5 glucose, and 50 sucrose) and cut into 350 μm coronal sections on a vibratome (Leica VT-1200S, Germeny). Slices containing the ACC were recovered for 1 h at 37 ℃ in oxygenated artificial cerebral spinal fluid (ACSF) containing (mM) 126 NaCl, 2.5 KCl, 1.2 NaHP_2_O_4_, 26 NaHCO_3_, 1.25 MgCl_2_, 2 CaCl_2_, and 10 glucose.

### Electrophysiological recording

For whole-cell recording, ACC slices were transferred to a recording chamber, and the ACSF with the aforementioned composition was continuously perfused 1–2 ml/min at 25 ℃ during the experiments (TC-324B, Warner, USA). PNs in the ACC layer V were visualized under infrared illumination using a fixed-stage upright microscope equipped with a 40X water-immersion lens (FN1, Nikon, Japan) and an IR CCD camera (DAGE-MTI). Recordings were obtained through the Multiclamp 700B patch-clamp amplifier and Digidata 1550A digitizer. Signals were sampled at 10 kHz and filtered at 1 kHz. The pipette had tip resistances of 3–5 MΩ.

For voltage-clamp recording, a borosilicate glass recording pipette was filled with internal solution containing (mM) 100 Cs-glucaric acid, 20 KCl, 10 HEPES, 4 Mg_2_ATP, 0.3 NaGTP, 10 Na-phosphocreacine and 3 QX-314 (pH 7.2 with, 280–300 mOsm). For sIPSC recording, normal ACSF solution was supplemented with 50 μM D-2-amino-5-phosphonovalerate (D-APV), and the voltage was clamped to 10 mV.

For current-clamp recording, a borosilicate glass recording pipette was filled with internal solution containing (mM) 110 K-gluconate, 20 KCl, 4 Mg_2_ATP, 10 HEPES, 0.3 NaGTP and 10 Na-phosphocreatine (pH 7.2 with, 280–300 mOsm). For current injection, PNs in the ACC layer V were current clamped and -200 pA-270 pA hyper- and depolarizing current was injected for 500 ms in 10 pA steps.

To verify whether PNs are influenced by hM3Dq- or hM4Di-transduced GABAergic interneuron cells, ACC slices that had been injected with AAV virus were prepared. CNO (10 μmol/L) was added to normal ACSF during recording.

In some neurons, an internal solution containing 0.2% biocytin was used. After recording, brain slices were immediately fixed in 4% paraformaldehyde in 0.1 M phosphate buffer saline (PBS, pH 7.4) for 12 h at 4 ℃. Slices were then washed in 0.01 M PBS containing 0.3% Triton X-100 (PBS-Triton). After this, sections were incubated in PBS-Triton containing 10% normal goat serum 1 h. Then, tissues were incubated in FITC-conjugated streptavidin for 48 h at 4 ℃. The immunofluorescence-labeled sections were then rinsed in PBS, mounted onto glass slides, air-dried, cover-slipped, and observed with a fluorescence microscope (Zeiss, Germany).

### Statistical analysis

The results are presented as the mean ± S.E.M and were analyzed by SPSS 20.0. For the experiments investigating behavioral responses over time or two factor designs, we used a two-way ANOVA, followed by Bonferroni tests to detect differences between the groups. For single independent factor designs, these experiments were analyzed using an unpaired Student’s* t* test. Statistical significance was set at *P* < 0.05.

## Results

### CFA rats displayed anxiety-like behaviors

**Fig. 1:**
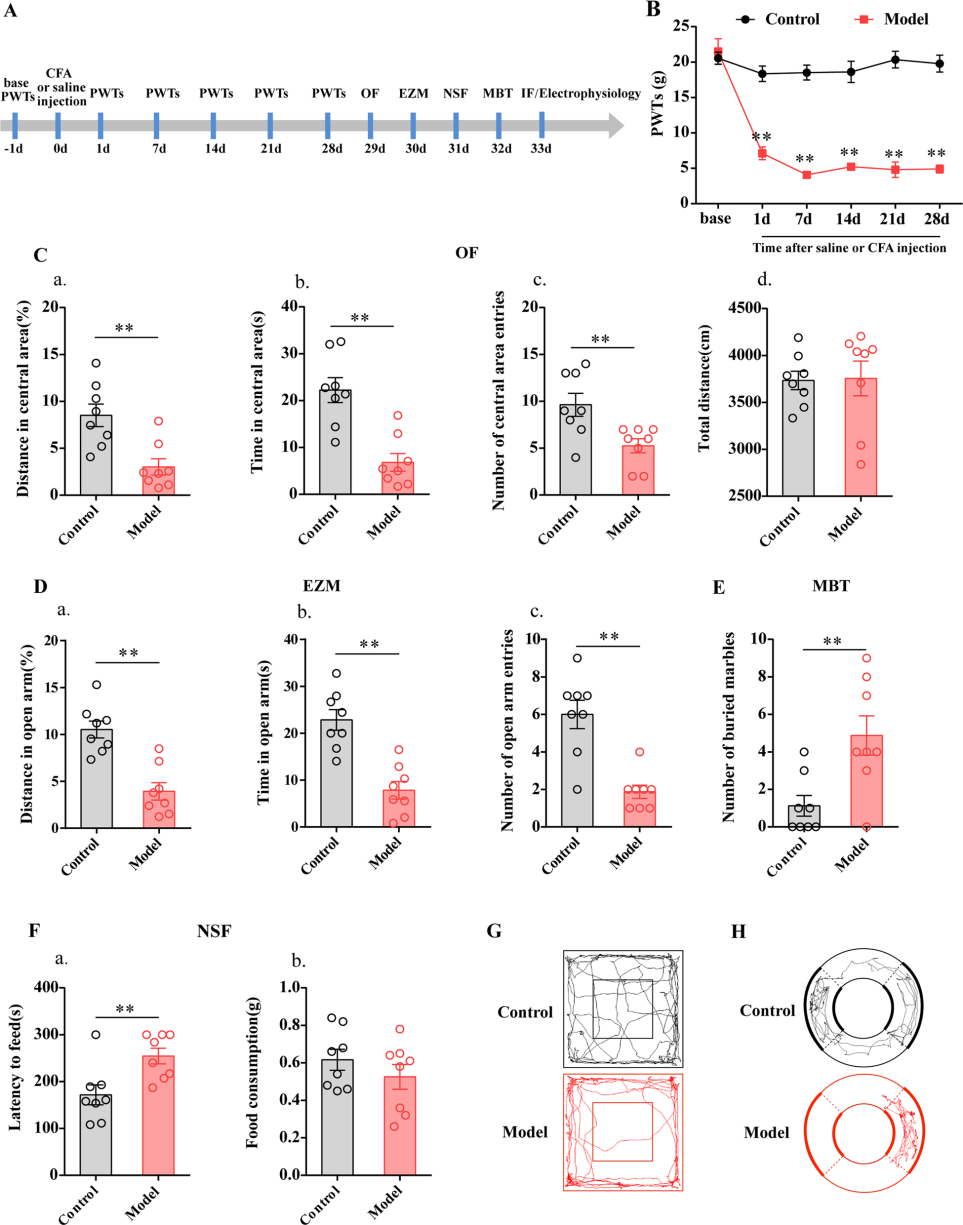
Sensory and affective characterization of chronic inflammatory pain rats. **A** Schematic of the experimental timeline. **B** PWTs of rats that received CFA injections. **C** Quantification of behavioral parameters in the OF. (a) The percentage of distance in the central zone, (b) time in the central zone, (c) the number of entries into the central zone, (d) and the total distance traveled throughout the arena of the control group and model group. **D** Quantification of behavioral parameters in the EZM. (a) the percentage of distance in the open arm, (b) time in the open arm, (c) the number of entries into the open arm of the control group and model group. **E** Quantification of behavioral parameters in the MBT. **F** Quantification of behavioral parameters in the NSF. (a) the time of latency to feed, (b) and the food consumption. The trajectories of rats in the control group and model group in the OF **G** on day 29 and EZM (H) on day 30d after CFA injection. All data represent the mean ± SEM, n = 8. **P < 0.01, compared to Control group

Rats injected with CFA exhibited anxiety-like behaviors 28 days or later [21, 22]. In this study, we observed pain-related behavior at base and 1 days, 7 days, 14 days, 21 days and 28 days after the injection according to PWTs and anxiety-like behavior from 29 to 32 days according to OF, EZM, NSF and MBT results (Fig. 1A).

Before injecting the CFA, PWTs between Control group and Model group were not remarkably different. PWTs of Model group rats were significantly decreased at 1 days, 7 days, 14 days, 21 days and 28 days after CFA injection (*P* < 0.01, Fig. 1B).

Twenty-eight days after CFA injection, the rats displayed multiple anxiety-like behaviors, including in the OF test (decreased percentage of distance traveled in the central area, time spent in the central area, and number of central area entries) (*P* < 0.01, Fig. 1C), the EZM test (decreased percentage of distance traveled in the open arm, time spent in the open arm, and number of open arm entries) (*P* < 0.01, Fig. 1D), the NSF test (increased latency to feed) (*P* < 0.01, Fig. 1F), and the MBT (increased number of buried marbles) (*P* < 0.01, Fig. 1E).

As a control for behavioral tests, we assessed the locomotor activity of control and model animals in the OF after 29 d after injection and confirmed our previous report [23] by showing that locomotor activity was not significantly affected (*P* > 0.05, Fig. 1Cd).

### Increased excitability of ACC PNs in the rat model of chronic inflammatory pain-induced anxiety rat

It has been reported that long-term synaptic plasticity in the ACC is important for chronic inflammatory pain management and pain-related anxiety [24]. In this study, we performed an IF experiment to examine the expression of c-Fos (a marker of neuron activation) and CaMKII (a marker of glutamatergic neurons) positive cells in the ACC and made patch-clamp electrophysiological recordings in acute ACC slices to investigate the excitability of ACC PNs.

**Fig. 2:**
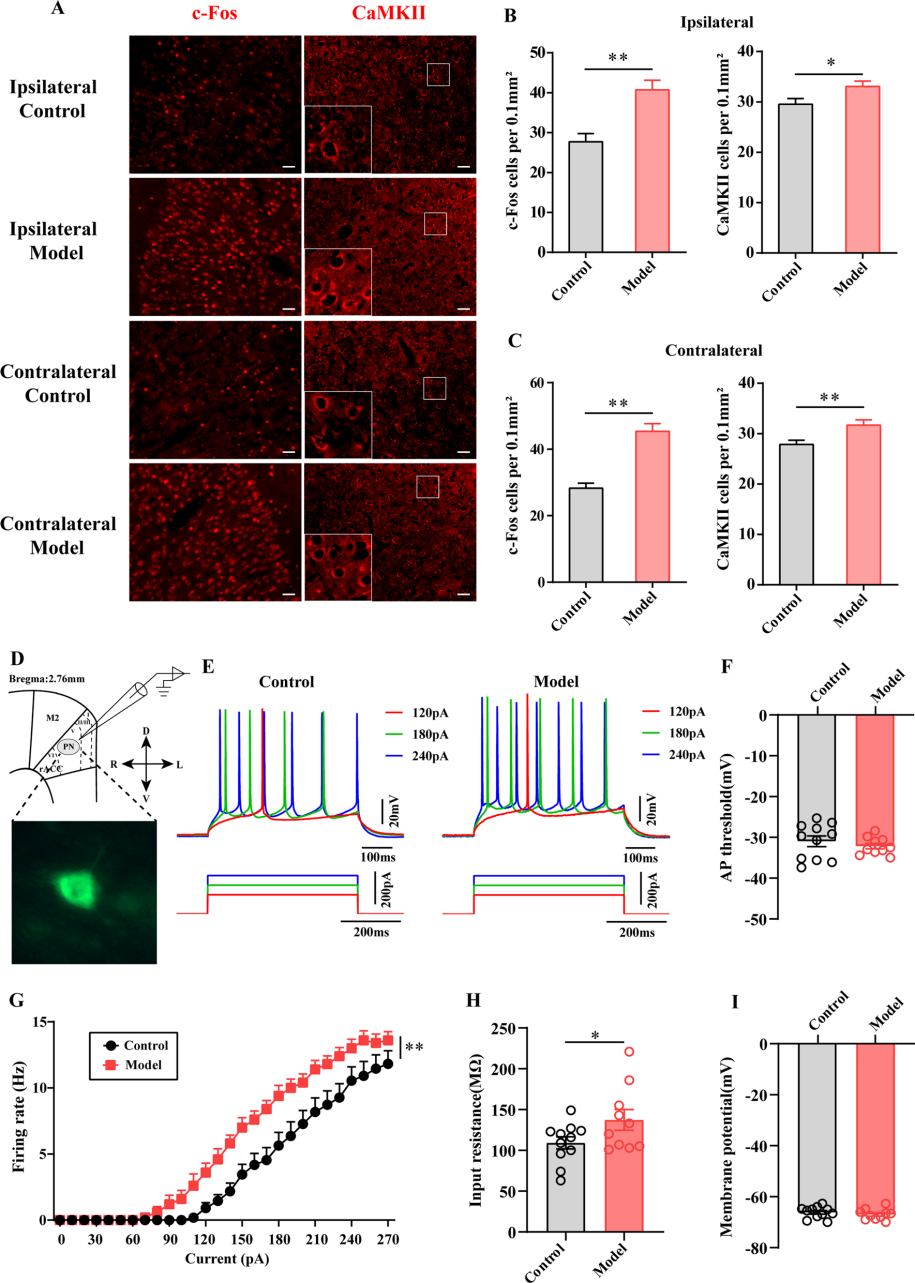
Chronic inflammatory pain-induce anxiety rat increased the excitability of ACC PNs **A** Representative images of c-Fos positive cells (red) and CaMKII (red) positive cells in the bilateral ACC of Control and Model group. The number of c-Fos positive cells and CaMKII positive cells per 0.1 mm^2^ in the ipsilateral ACC (**B**) and contralateral ACC (**C**) of Control and Model group. **D** Schematic diagram depicting the anatomical localization in electrophysiological recording and the morphological property of pyramidal neurons. n = 3–4. *P < 0.05, **P < 0.01, compared to the Control group, Bar = 50 μm. **E** The firing phenotypes of PNs in ACC layer V in Control and Model groups **G** The firing rates in response to prolonged depolarizing current injections of increasing amplitudes in control and model groups. The input resistance (**H**), AP threshold (**F**) and resting membrane potentials (**I**) of PNs in ACC layer V were statistically analyzed in control and model groups. n = 9 or 12 neurons from 3 or 4 rat/group. *P < 0.05, **P < 0.01, compared to the Control group. All data represent the mean ± SEM

Both ipsilateral and contralateral c-Fos- and CaMKII-positive cells in the ACC were significantly increased (Fig. 2A–C).

The morphological properties of the evaluated neurons in the ACC layer V are shown in Fig. 2D. Similar proportions of the different firing types were found in both Control and Model group, demonstrating that chronic inflammatory pain-induced anxiety in rats did not affect the firing phenotypes of ACC PNs (Fig. 2E). Notably, however, firing rates in response to prolonged depolarizing current injections of increasing amplitudes were significantly larger in ACC PNs from model rats than in those from control rats (*P* < 0.01, Fig. 2G). The increased excitability of ACC PNs in model rats was accompanied by increases in input resistance (*P* < 0.05, Fig. 2H). Importantly, the AP threshold and resting membrane potentials of ACC PNs were not altered in Model group compared to that of Control group (*P* > 0.05, Fig. 2FI). Together with the results of c-Fos and CaMKII immunohistochemistry, these electrophysiological experiments suggested that hyperexcitability of ACC PNs underlies chronic inflammatory pain-induced anxiety.

### Reduction of inhibitory presynaptic transmission in the ACC in the rat model of chronic inflammatory pain-induced anxiety

**Fig. 3:**
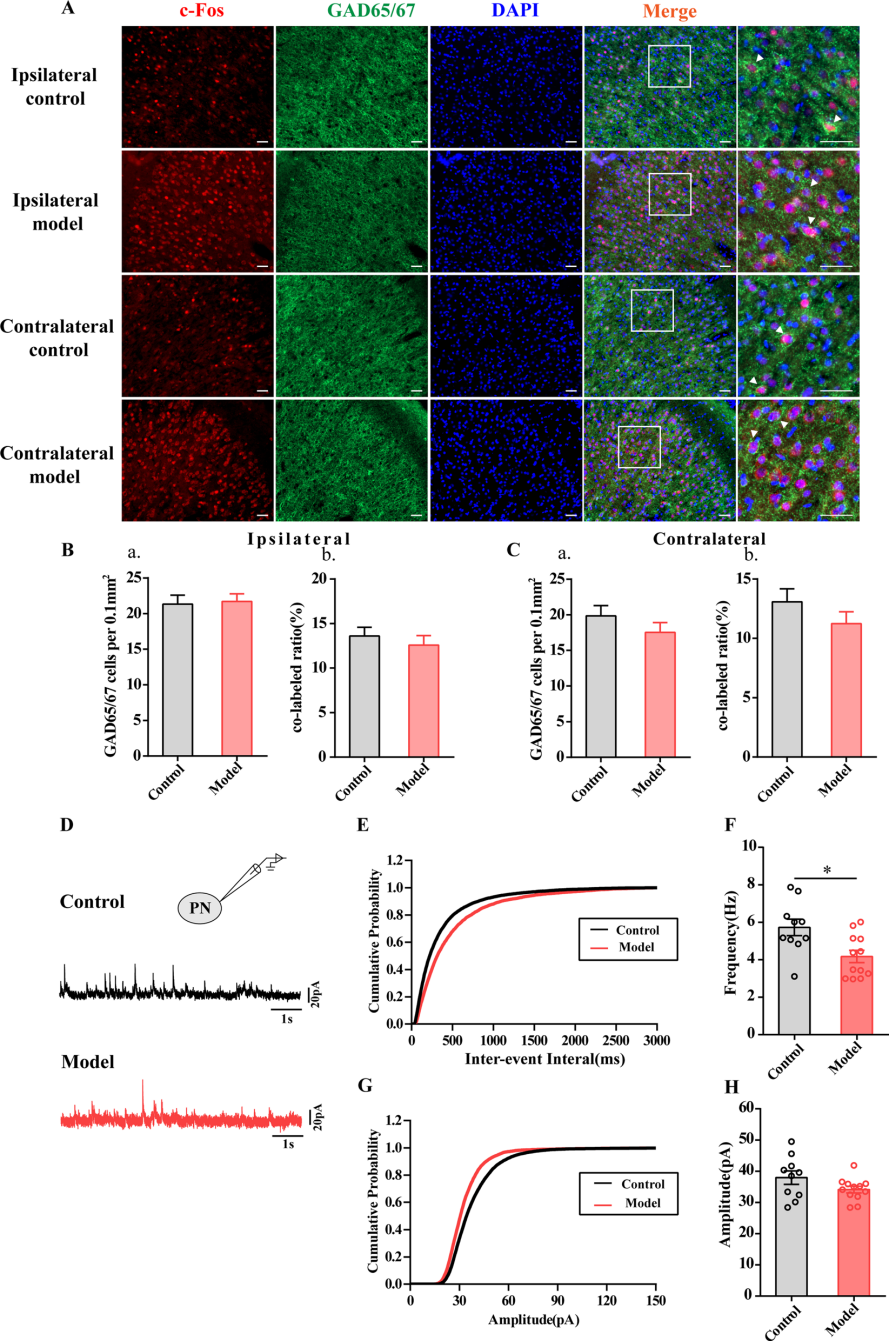
Chronic inflammatory pain rats reduced the inhibitory presynaptic transmission in the ACC. **A** Representative images with immunohistochemical staining for c-Fos (red) and GAD65/67 (Green), and the presentative images of c-Fos merged with GAD65/67 in the bilateral ACC of Control and Model groups. The number of GAD65/67 cells per 0.1 mm^2^ and the co-labeled ratio in the ipsilateral ACC (**B**) and contralateral ACC (**C**) of control and model group rats. n = 3–4. *P < 0.05, compared to the Control group. Bar = 50 μm. **D** The representative showed sIPSCs of PNs in ACC layer V in each group. The cumulative frequency (**E**) and amplitude (**G**) histogram of sIPSCs in different groups. The average frequency (**F**) and amplitude (**H**) of sIPSCs were statistically analyzed in Control and Model groups. n = 9 or 12 neurons from 3 or 4 rat/group. *P < 0.05, compared to the Control group. All data represent the mean ± SEM,

Glutamic acid decarboxylase (GAD) is localized only in GABAergic presynaptic neurons and terminals in two common forms, GAD65 and GAD67; these forms are responsible for synthesizing GABA [25]. We used IF to observe the expression of GAD65/67 in cells and coexpression with c-Fos. Neither the number of GAD65/67-positive cells or their coexpression with c-Fos in the bilateral ACC was decreased in Control group and Model group (*P* > 0.05, Fig. 3A–C). Therefore, the synthesis of GABA may not be associated with chronic inflammatory pain-induced anxiety in rats.

The subtypes of GABAergic neurons mainly include parvalbumin (PV), somatostatin (SST), vasoactive intestinal peptide (VIP), and neuropeptide Y (NPY) [26]. Our previous paper has been observed that only PV interneurons were involved in chronic inflammatory pain related anxiety-like behaviors and pain sensation [27]. We observed the expression of PV positive cells and its coexpression with c-Fos. The number of PV positive cells in the bilateral ACC was decreased in Model group compared with that in Control group (Additional file 1: Fig. S1BC). There were not significant different between the Control group and Model group about the number of co-expression of PV and c-Fos positive cells in the bilateral ACC (sFig.1BC). In addition, we also further analyzed the number of PV positive cells in the ACC layer V. The number of PV positive cells in the bilateral ACC layer V was decreased in the model rats (Additional file 1: Fig. S1DE). However, similarly the previous results, the number of c-fos-IR positive cells that label by PV was not changed (Additional file 1: Fig. S1DE).

Then, we performed whole-cell patch-clamp recordings to record sIPSCs in ACC PNs layer V in voltage-clamp mode at 10 mV. The electrophysiological properties of the neurons are shown in Fig. 3D. The tissue was perfused with 6-cyano-7-nitroquinoxaline-2,3-dione, an a-amino-3-hydroxy-5-methyl-4-isoxazolepropionic acid/KA receptor antagonist, AP-V, and an N-methyl-d-aspartic acid receptor antagonist. We recorded 10 neurons from 3 control animals and found that the average frequency and amplitude of sIPSCs was 5.72 Hz and 37.96 pA, respectively (Fig. 3F, H). In contrast, in 12 neurons recorded from 4 model animals, the average frequency and amplitude of sIPSCs was 4.17 Hz and 34.12 pA, respectively (Fig. 3F, H). The results showed that the average frequency of sIPSCs in Model group was decreased compared with that in Control group (*P* < 0.01). However, the average amplitude of sIPSCs was not altered between Control and Model group (*P* > 0.05). Therefore, we speculated that a reduction in GABA release resulted in a decrease in inhibitory presynaptic transmission and may be involved in chronic inflammatory pain-related anxiety.

### ACC GABAARs are involved in chronic inflammatory pain-induced anxiety processing

GABA, the primary mediator of inhibitory neurotransmission, is involved in the change in excitation in ACC neurons [28]. GABA acts through ionotropic A- and metabotropic B-type receptors and plays a key role in the brain’s function. GABA_A_ receptors are crucial to inhibiting brain excitability, and GAB_A_B receptors mainly modulate the generation of excitatory postsynaptic potentials and long-term potentiation [13]. To understand whether the GABAergic system is involved in chronic inflammatory pain and pain-related anxiety, we microinjected the GABA_A_R agonist muscimol into the ACC of rats and observed its effect on anxiety behaviors.

**Fig. 4:**
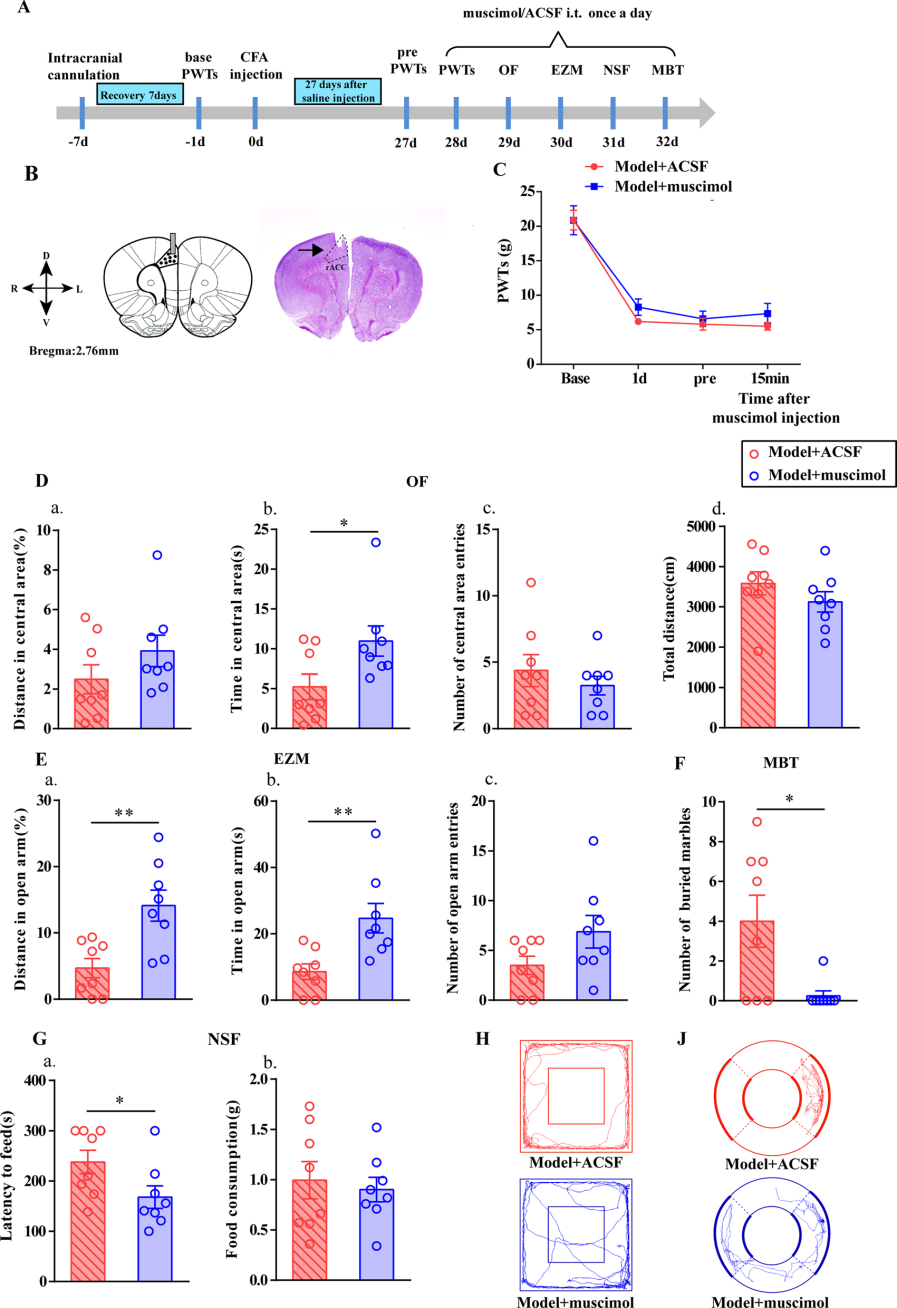
Intra-ACC injection of GABA_A_R agonist induced anxiety-like behavior. **A** A schematic of the experimental design. **B** Representative figures show the anatomical localization of the rACC. **C** PWTs of model rats that received muscimol (GABA_A_R agonist) injections. **D** Quantification of behavioral parameters in the OF. (a) the percentage of distance in the central zone, (b) time in the central zone, (c) the number of entries into the central zone, (d) and the total distance traveled throughout the arena of the Model + ACSF group and Model + picrotoxin group. **E** Quantification of behavioral parameters in the in EZM. (a) the percentage of distance in the open arm, (b) time in the open arm, (c) the number of entries into the open arm of the Model + ACSF group and Model + picrotoxin group. **F** Quantification of behavioral parameters in the MBT. **G** Quantification of behavioral parameters in the NSF. (a) The time of latency to feed, (b) and the food consumption. The trajectories of rats in the Model + ACSF group and Model + picrotoxin group in the OF (**I**) and EZM (**J**). All data represent the mean ± SEM, n = 8. *P < 0.05, **P < 0.01, compared to the Model + ACSF group

Intra-ACC injection of muscimol increased the time Model + muscimol rats spent in central area in the OF test (*P* < 0.05, Fig. 4D) and their percentage of distance and time spent in the open arm in the EZM test (*P* < 0.01, Fig. 4E). In addition, it decreased the number of marbles Model + muscimol rats buried in the MBT (*P* < 0.05, Fig. 4F), in conjunction with a decrease in the latency to feed in the NSF test relative to that of the Model + ACSF animals (*P* < 0.05, Fig. 4G). As expected, muscimol did not affect locomotor activity (*P* > 0.05, Fig. 4Dd).

Furthermore, we determined the effect of the GABA_A_R antagonist picrotoxin by microinjecting it into the ACC of rats; then, these rats performed the same tests in normal rat. Interestingy, Control + picrotoxin animals exhibited a significant decrease in the percentage of distance traveled in, time spent in, and number of entries into the central area relative to the Control + ACSF animals in the OF test (*P* < 0.05, sFig. 2E). In addition, Control + picrotoxin animals spent less time in the open arm, had a lower percentage of distance traveled in the open arm, and had fewer entries into the open arm compared with the Control + ACSF rats in this test in the EZM (*P* < 0.05, Additional file 2: Fig. S2F). Furthermore, the number of marbles buried by Control + picrotoxin animals was increased relative to that of Control + ACSF animals (*P* < 0.05, Additional file 2: Fig. SG). However, in terms of latency to feed, Control + picrotoxin animals performed similarly to Control + ACSF animals (*P* > 0.05, Additional file 2: Fig. SH). Thus, GABA_A_R antagonism induced anxiety in normal rats. As expected, picrotoxin did not affect locomotor activity (*P* > 0.05, Additional file 2: Fig. SEd) or PWTs (*P* > 0.05, Additional file 2: Fig. SD).

### ACC GABAergic neurons are involved in chronic inflammatory pain-induced anxiety processing

The next set of experiments to determine whether activation of GABAergic neurons is sufficient to reverse chronic inflammatory pain and pain-induced anxiety-like behavior. To do this, we infused excitatory DREADD hM3Dq (AAV-) into the ACC of model rats and performed the same battery of behavioral tests after intraperitoneal injection of CNO (2 mg/kg body weight).

We used IF to observe the location and specificity of the virus, and the results showed that VGAT-mCherry and GAD65/67 were coexpressed and that VGAT-mCherry and CamkII were not coexpressed (Additional file 3: Fig. S3).

**Fig. 5:**
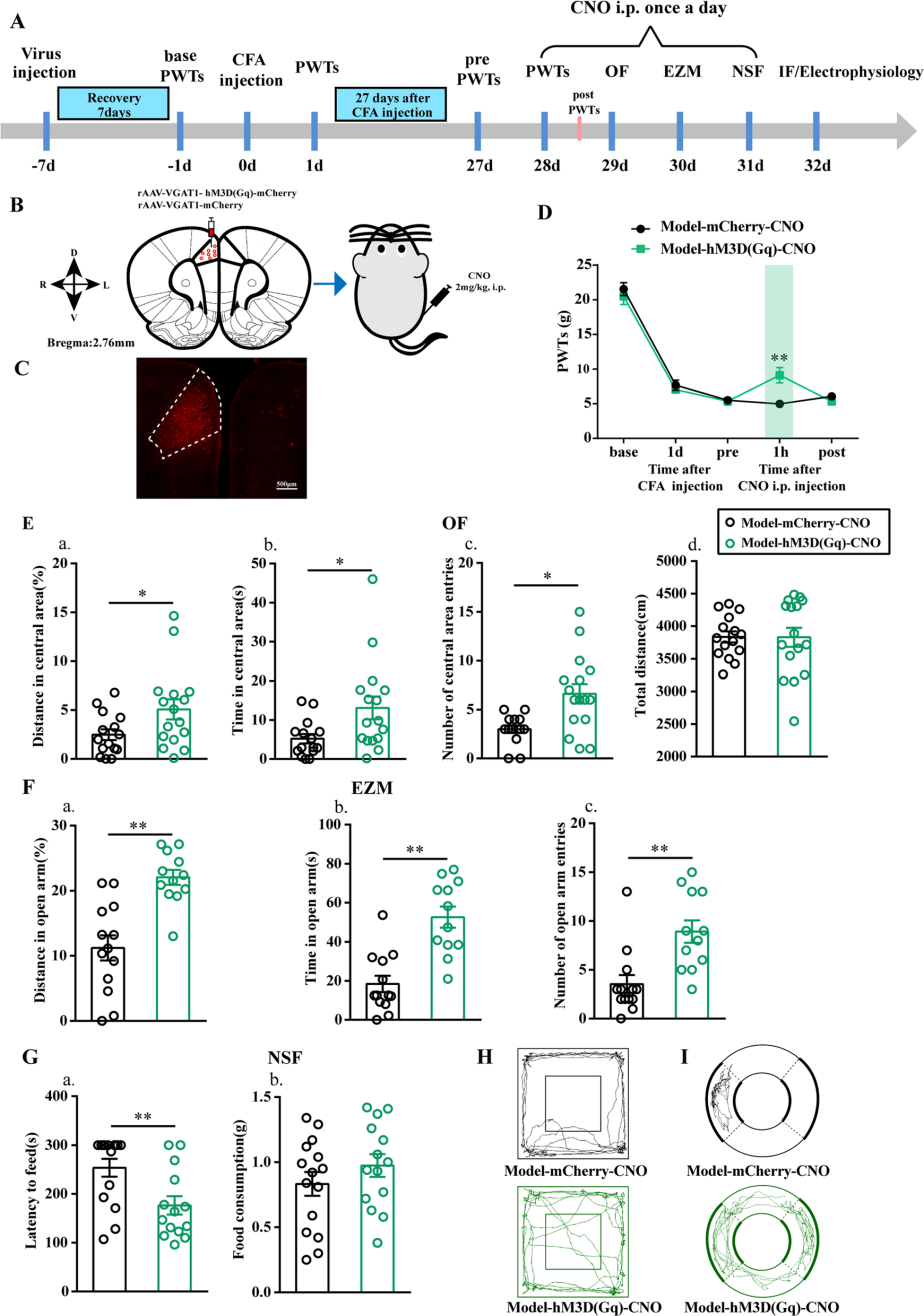
Chemogenetic activation of GABAergic neurons in the ACC alleviates chronic inflammatory pain and pain-induced anxiety-like behavior. **A** A schematic of the experimental design. **B** Microinjection of rAAV-VGAT1-hM3D(Gq)-mCherry or rAAV-VGAT1-mCherry into into the contralateral ACC of CFA rats, and intraperitoneal injections of CNO (2 mg/kg body weight) in Model-mCherry-CNO and Model-hM3D(Gq)-CNO rats before the behavioral test. **C** A representative figure showing expression of mCherry in the ACC. **D** PWTs changes of model rats with CNO-mediated chemogenetic activation of GABAergic neurons in the ACC (n = 16 in the Model-mCherry-CNO group; n = 16 in the Model-hM3D(Gq)-CNO group). **E** Quantification of behavioral parameters in the OF (n = 15 in the model-mCherry-CNO group; n = 16 in the model-hM3D(Gq)-CNO group). (a) The percentage of distance in the central zone, (b) time in the central zone, (c) the number of entries into the central zone, (d) and the total distance traveled throughout the arena of the Model-mCherry-CNO group and Model-hM3D(Gq)-CNO group. **F** Quantification of behavioral parameters in the EZM (n = 13 in the model-mCherry-CNO group; n = 12 in the model-hM3D(Gq)-CNO group). (a) The percentage of distance in the open arm, (b) time in the open arm, (c) the number of entries into the open arm of the Model-mCherry-CNO group and Control-hM3D(Gq)-CNO group. **G** Quantification of behavioral parameters in the NSF (n = 15 in the model-mCherry-CNO group; n = 14 in the model-hM3D(Gq)-CNO group). (a) The time of latency to feed, (b) and the food consumption. The trajectories of rats in the Model-mCherry-CNO group and Model–hM3D(Gq)-CNO group in the OF (H) and EZM (I). Bar = 500 μm. All data represent the mean ± SEM. *P < 0.05, **P < 0.01, compared to the Model-mCherry-CNO group

As illustrated in Fig. 5, PWTs were increased in model-hM3D(Gq)-CNO rats compared with those in model-mCherry-CNO rats (*P* < 0.01, Fig. 5D). As expected, CNO-mediated chemogenetic activation of GABAergic neurons in the ACC reduced pain-related anxiety behaviors of model rats. Model-hM3D(Gq)-CNO rats exhibited a significant increase in the percent of distance traveled in the central area, time spent in the central area, and the number of central area entries relative to the model-mCherry-CNO rats in the OF test (*P* < 0.05, Fig. 5E). In addition, model-hM3D(Gq)-CNO rats spent more time in the open arm in the EZM test, in conjunction with exhibiting an increased percent of distance traveled in the open arm and number of open area entries in this test compared with model-mCherry-CNO rats (*P* < 0.01, Fig. 5F). Furthermore, in terms of the latency to feed in the NSF test, model-hM3D(Gq)-CNO rats showed a significant decrease compared with model-mCherry-CNO rats (*P* < 0.01, Fig. 5G). Importantly, locomotor activity between model-hM3D(Gq)-CNO rats and model-mCherry-CNO rats was not significantly affected (*P* > 0.05, Fig. 5Ed).

Furthermore, we stereotaxically injected rAAV-VGAT1-hM4(Di)-mCherry into the bilateral ACC of control rats (Additional file 4: B) to observe the effect of GABAergic interneurons in the ACC on pain and anxiety-like behavior.

The effects of selective inhibition of GABAergic neurons were then measured after i.p. injection of CNO (2 mg/kg body weight) in Control-mCherry and Control-hM4D(Gi) rats. As illustrated in Additional file 4: Fig. S4D-G, CNO-mediated chemogenetic inhibition of GABAergic neurons in the ACC induced anxiety-like behavior but had no effect on pain (*P* > 0.05). For example, in the open field test (Additional file 4: E), the percent of distance traveled in central area, time spent in the central area, and number of central area entries by control-hM4D(Gi) rats were significantly reduced compared with control-mCherry rats (*P* < 0.05); in elevated zero maze test (Additional file 4: F), the percent of distance traveled in the open arm, time spent in the open arm, number of open area entries by control-hM4D(Gi) rats were significantly reduced compared with control-mCherry rats (*P* < 0.01); in novelty-suppressed feeding test (Additional file 4: G), latency to feed in control-hM4D(Gi) rats was significantly increased compared with control-mCherry rats (*P* < 0.05).

Importantly, locomotor activity between control-hM4D(Gi) rats and control-mCherry rats was not significantly affected (*P* > 0.05, Additional file 4:  4Ed).

### Chemogenetic activation of GABAergic neurons in the ACC decreased the excitability of ACC pyramidal neurons and enhanced inhibitory presynaptic transmission

**Fig. 6:**
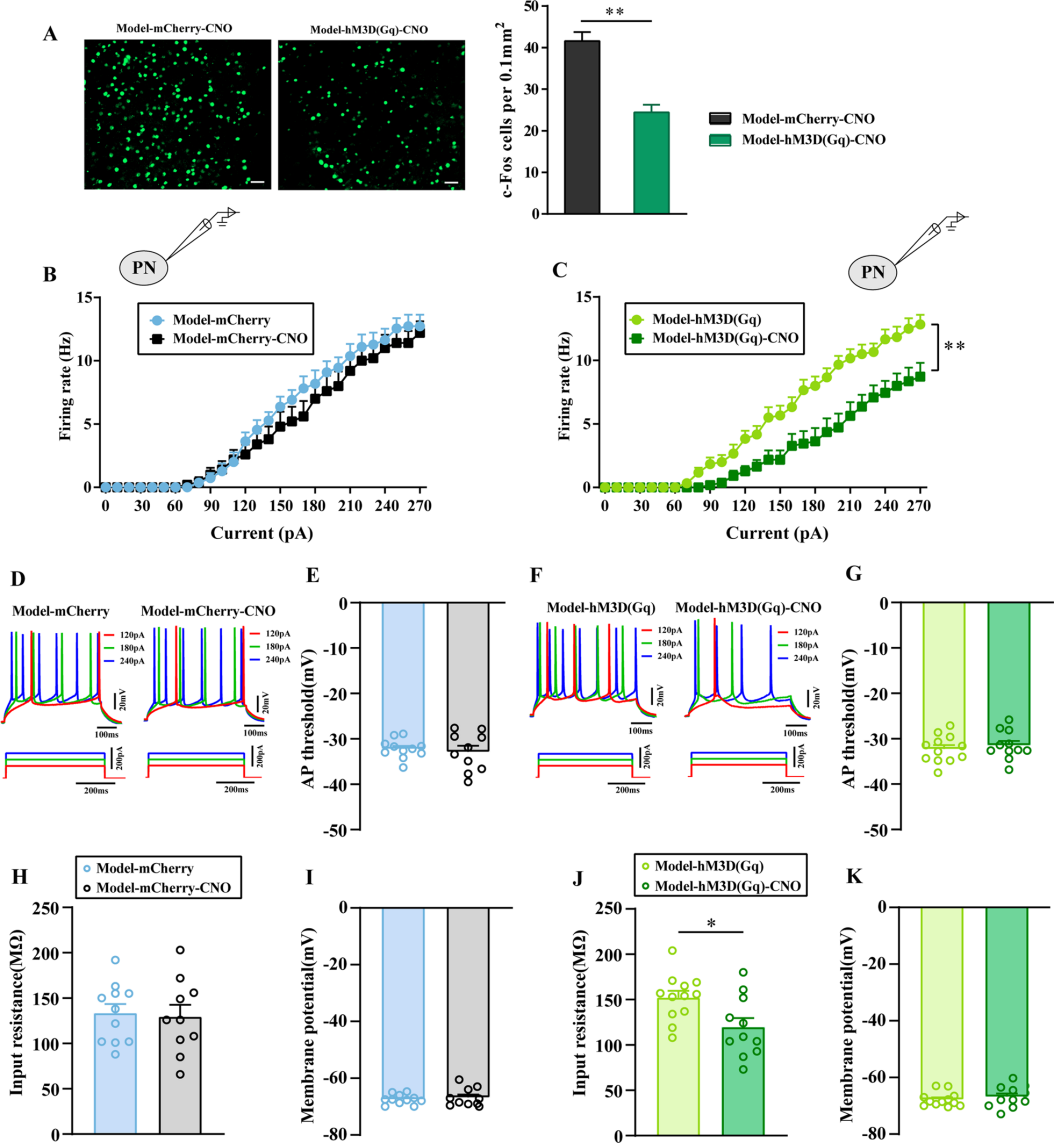
Chemogenetic activation of GABAergic neurons in the ACC decreased the excitability of ACC PNs. **A** Representative images of c-Fos in the contralateral ACC of Model-mCherry-CNO and Model-hM3D(Gq)-CNO group rats. The number of C-Fos cells per 0.1mm^2^ in the contralateral ACC of Model-mCherry-CNO and Model-hM3D(Gq)-CNO group rats. n = 3–4. **P < 0.01, Model-hM3D(Gq) group vs Model-hM3D(Gq)-CNO group. Bar = 50 μm. **B**, **C** The firing rates in response to prolonged depolarizing current injections of increasing amplitudes in different groups. **D**, **F** The firing phenotypes changes of CNO application of PNs in ACC layer V. The input resistance (**H**, **J**), AP threshold (**E**, **G**) and membrane potentials (**I**, **K**) of PNs in ACC layer V were statistically analyzed in each group. n = 10 neurons from 4 rat/group. **P < 0.01, Model-hM3D(Gq) group vs Model-hM3D(Gq)-CNO group. All data represent the mean ± SEM

Then, we used IF to observe the expression of c-Fos in cells to determine whether the activation of GABAergic neurons could modulate the activity of ACC neurons. As shown in Fig. 6A, cells expressing of c-Fos in the contralateral ACC of the Model-hM3D(Gq)-CNO group were significantly decreased compared with that of the Model-mCherry-CNO group (*P* < 0.01), which indicated that activation of GABAergic neurons could decrease ACC excitability.

To further determine the relationship between GABAergic neurons and the excitability of ACC PNs, we performed whole-cell patch-clamp recordings.

Similar proportions of the different firing types were found in the Model-mCherry and Model-hM3D(Gq) groups with and without CNO application, demonstrating that CNO perfusion did not affect the firing phenotypes of ACC PNs (Fig. 6D, F). The firing rate, input resistance, AP threshold and membrane potentials of ACC neurons did not differ between the Model-mCherry group with and without CNO perfusion (*P* > 0.05, Fig. 6B, E, H, I). Interestingly, firing rates in response to prolonged depolarizing current injections of increasing amplitudes were significantly smaller in ACC PNs with CNO application that in those without CNO application in the Model-hM3D(Gq) group (*P* < 0.01, Fig. 6C). With CNO perfusion, the decreased excitability of ACC PNs in model-hM3D(Gq) rats was accompanied by decreases in input resistance (*P* < 0.05, Fig. 6J). Importantly, the AP threshold and resting membrane potentials of ACC PNs were not altered between the Model-hM3D(Gq) group and Model-hM3D(Gq)-CNO group (*P* > 0.05, Fig. 6G, K).

Furthermore, to clarify the relationship between GABA release and the excitability of ACC PNs, we measured sIPSCs of ACC PNs with and without CNO perfusion.

**Fig. 7:**
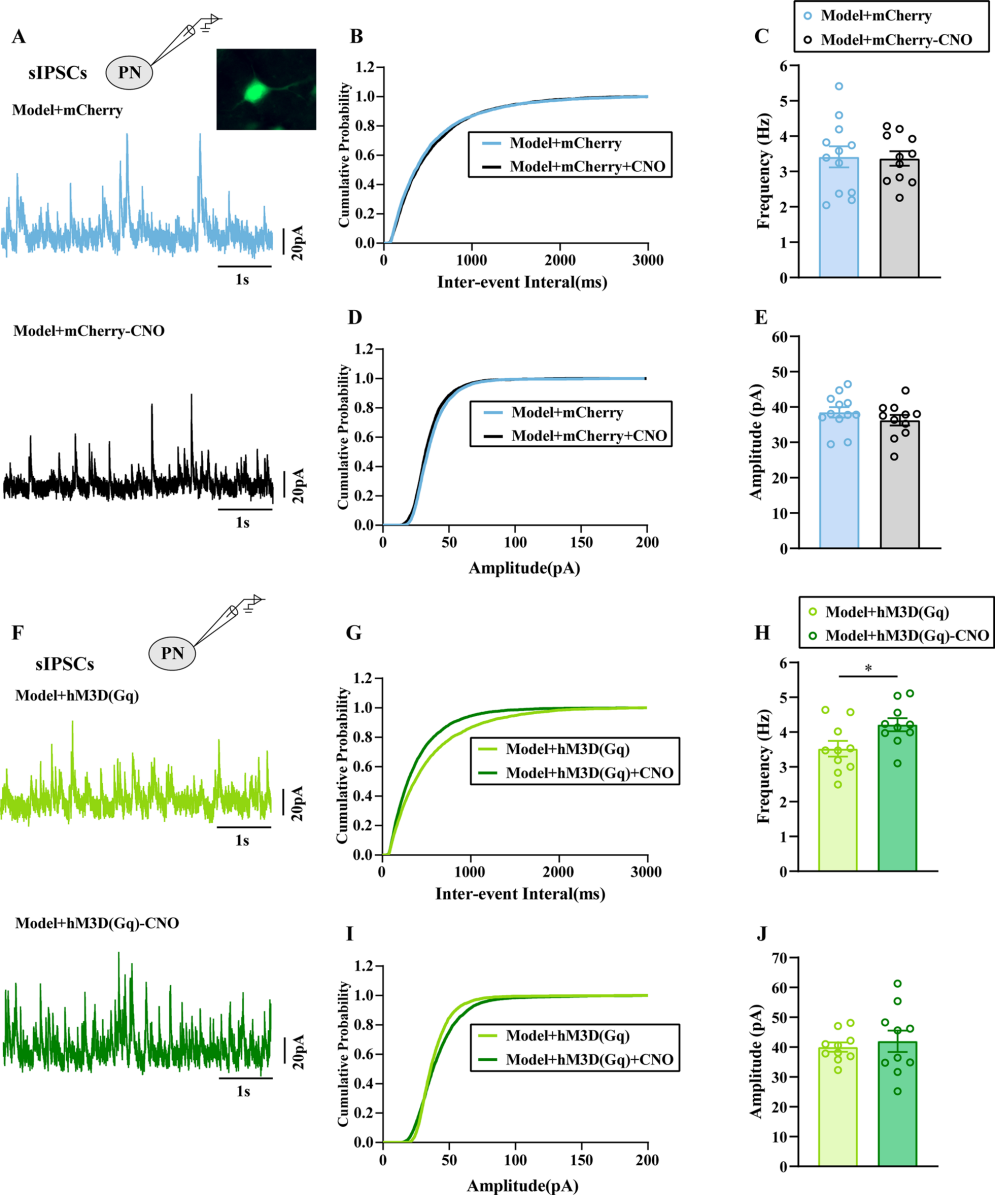
Chemogenetic activation of GABAergic neurons in the ACC enhanced the inhibitory presynaptic transmission. **A**, **F** The representative showed sIPSCs of PNs in ACC layer V in each group. The cumulative frequency (**B**, **G**) and amplitude (**D**, **I**) histogram of sIPSCs in different groups. The average frequency (**C**, **H**) and amplitude (**E**, **J**) of sIPSCs were statistically analyzed in each group. All data represent the mean ± SEM, n = 10 neurons from 4 rat/group. *P < 0.05, Model-hM3D(Gq) group vs Model-hM3D(Gq)-CNO group

The electrophysiological and morphological properties of the evaluated neurons in the ACC layer V are shown in Fig. 7A, F. The tissue was perfused with AP-V. We recorded 10 neurons from 4 model-mCherry animals without CNO application and found that the average frequency and amplitude of sIPSCs was 3.414 Hz and 38.51 pA, respectively (Fig. 7C, E). In contrast, in 10 neurons recorded from 4 model-mCherry animals with CNO application, the average frequency and amplitude of sIPSCs was 3.368 Hz and 36.27 pA, respectively (Fig. 7C, E). The results showed that neither the average frequency nor the amplitude of sIPSCs was altered with or without CNO perfusion in the Model-mCherry group (*P* > 0.05). We also observed sIPSC in the Model-hM3D(Gq) group with or without CNO application. The average frequency and amplitude of sIPSCs was 3.521 Hz, 39.99 pA respectively, based on recordings 10 neurons from 4 model-hM3D(Gq) animals without CNO application (Fig. 7H, J). In contrast, in 10 neurons recorded from 4 model-hM3D(Gq) animals with CNO application, the average frequency and amplitude of sIPSCs was 4.21 Hz, 41.95 pA, respectively (Fig. 7H, J). These results suggested that the average frequency of sIPSCs in the modell-hM3D(Gq) group with CNO application was increased compared with that in the group without CNO applicatin (*P* < 0.05, Fig. 7H). However, the average amplitude of sIPSC was not altered (*P* > 0.05, Fig. 7J).

### Chemogenetic inhibition of GABAergic neurons in the ACC enhanced the excitability of ACC pyramidal neurons and reduced inhibitory synaptic transmission

Then, we used IF to observe the expression of c-Fos in cells to determine whether the inhibition of GABAergic neurons could modulate the activity of ACC neurons. As shown in Fig. 7A, B, the number of cells expressing c-Fos in the bilateral ACC of the Control-hM4D(Gi)-CNO group was significantly increased compared with that of the control-mCherry-CNO group (*P* < 0.01), which indicated that inhibition of GABAergic neurons could increase ACC excitability.

To further determine the relationship between GABAergic neurons and the excitability of ACC PNs, we performed whole-cell patch-clamp recordings.

**Fig. 8:**
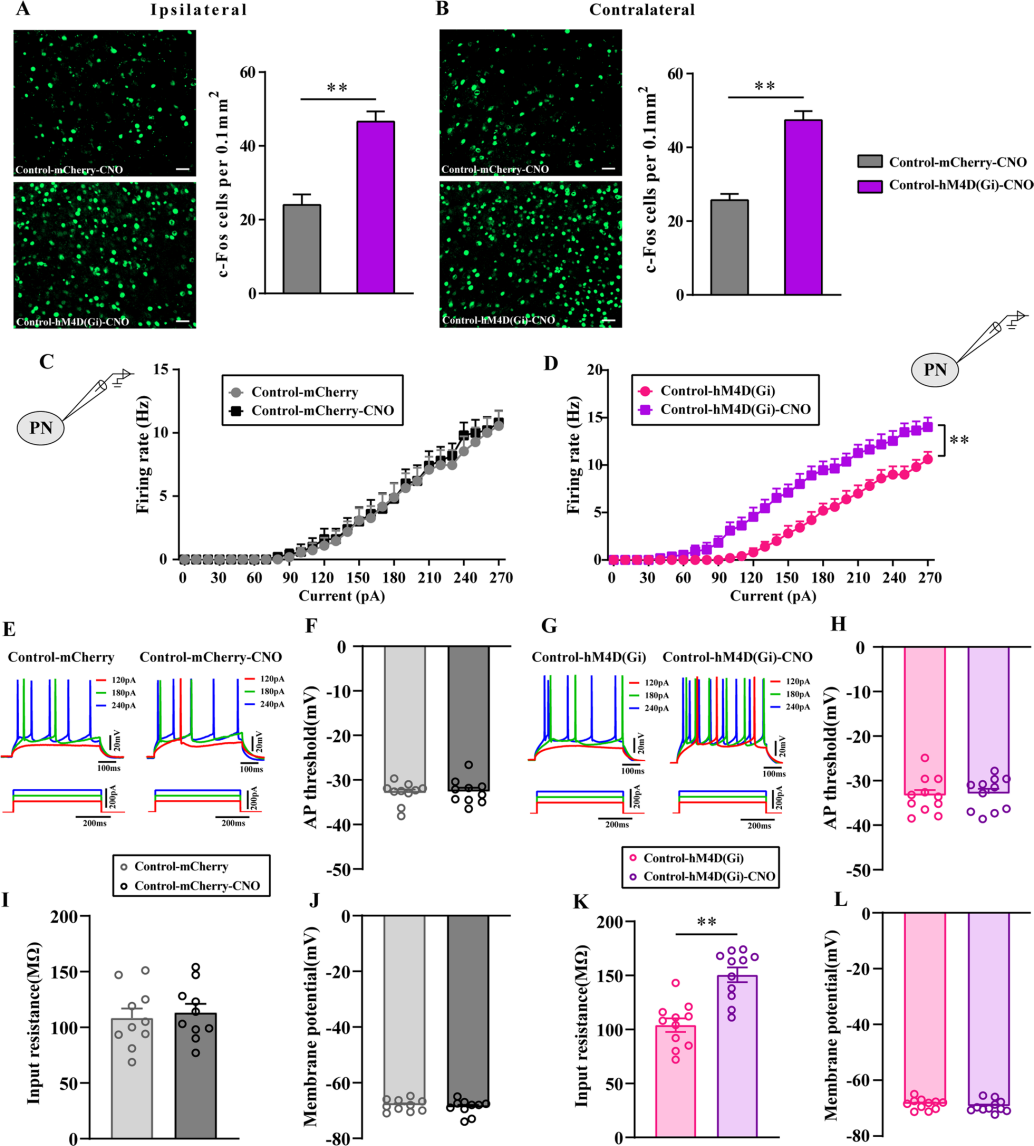
Chemogenetic inhibition of GABAergic neurons in the ACC enhanced the excitability of ACC PNs. The number of c-Fos cells per 0.1mm^2^ in the ipsilateral ACC (**A**) and contralateral ACC (**B**) of Control-mCherry-CNO and Control-hM4D(Gi)-CNO rats. n = 3–4. **P < 0.01, Control-hM4D(Gi) group vs Control-hM4D(Gi)-CNO group. **C**, **D** The firing rates in response to prolonged depolarizing current injections of increasing amplitudes in different groups. **E**, **G** The firing phenotypes changes of CNO application of PNs in ACC layer V. The input resistance (**I**, **K**), AP threshold (**F**, **H**) and membrane potentials (**J**, **L**) of PNs in ACC layer V were statistically analyzed in each group. Bar = 50 μm. n = 10 neurons from 5 rat/group. **P < 0.01, Control-hM4D(Gi) group vs Control-hM4D(Gi)-CNO group. All data represent the mean ± SEM

Similar proportions of the different firing types were found in the Control-mCherry and Control-hM4D(Gi) groups with and without CNO application, demonstrating that CNO perfusion did not affect the firing phenotypes of ACC PNs (Fig. 8E, G). the firing rate, AP threshold, input resistance and membrane potentials of ACC neurons were not altered in the Control-mCherry group with or without CNO perfusion (*P* > 0.05, Fig. 8C, F, I, J). Interestingly, firing rates in response to prolonged depolarizing current injections of increasing amplitudes were significantly larger in ACC PNs with CNO application than in those without CNO application in the Control-hM4D(Gi) group (*P* < 0.01, Fig. 8D). With CNO perfusion, the increased excitability of ACC PNs from control-hM4D(Gi) rats was accompanied by increases in input resistance (*P* < 0.05, Fig. 8K). Importantly, the AP threshold and resting membrane potentials of ACC PNs did not differ between the control-hM4D(Gi) group and the Control-hM4D(Gi)-CNO group (*P* > 0.05, Fig. 8HL).

Furthermore, to clarify the relationship between GABA release and the excitability of ACC PNs, we measured sIPSCs of ACC PNs with and without CNO perfusion.

**Fig. 9:**
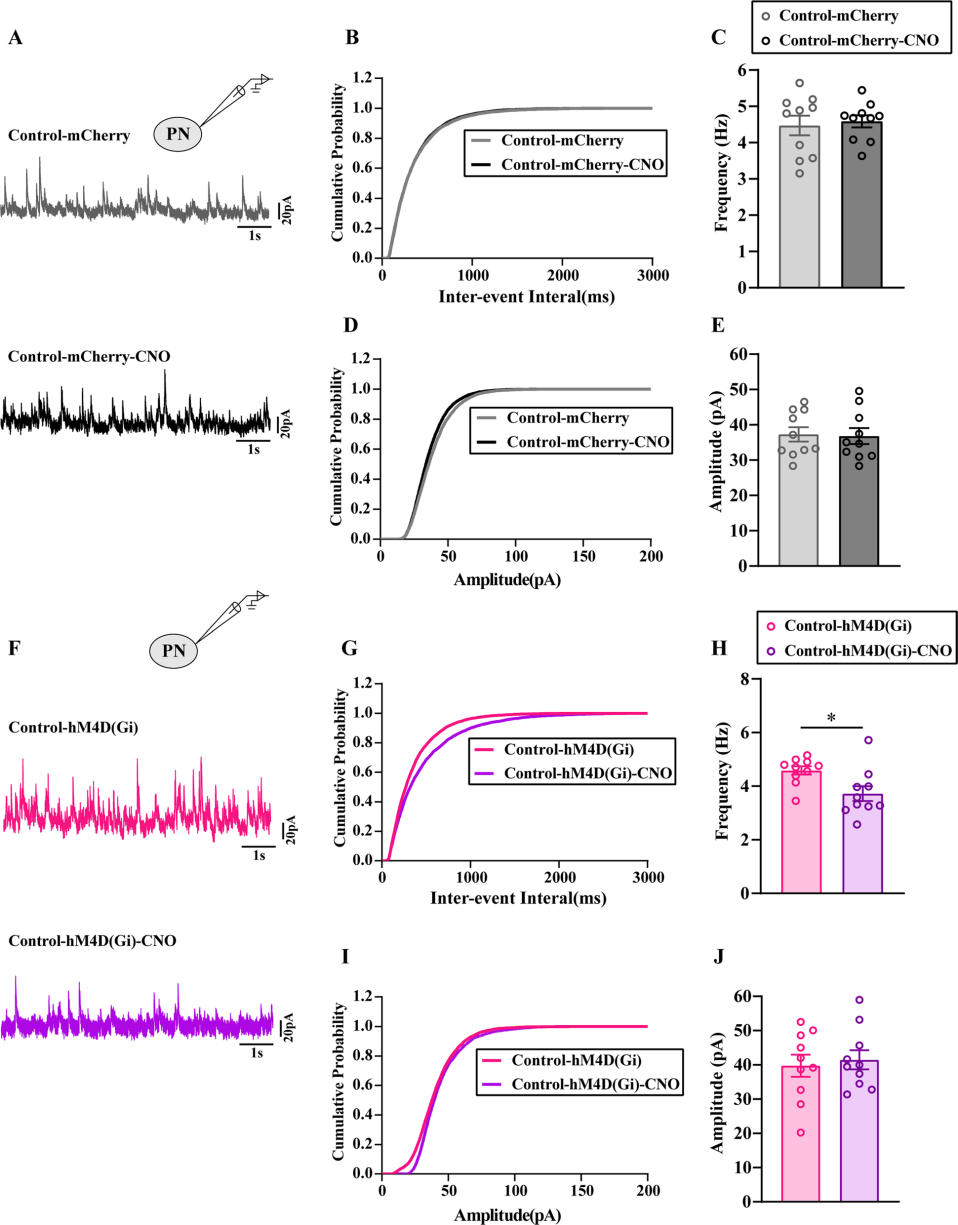
Chemogenetic inhibition of GABAergic neurons in the ACC reduced the inhibitory presynaptic transmission. **A**, **F** The representative showed sIPSCs of PNs in ACC layer V in each group. The cumulative frequency (**B**, **G**) and amplitude (**D**, **I**) histogram of sIPSCs in different groups. The average frequency (**C**, **H**) and amplitude (**E**, **J**) of sIPSCs were statistically analyzed in each group. All data represent the mean ± SEM, n = 10 neurons from 5 rat/group. *P < 0.05, Control-hM4D(Gi) group vs Control-hM4D(Gi)-CNO group

The electrophysiological properties of the evaluated neurons are shown in Fig. 9A, F. The tissue was perfused with AP-V. We recorded 10 neurons from 4 control-mCherry animals without CNO application and found that the average frequency and amplitude of sIPSCs was 37.32 Hz and 4.47 pA, respectively (Fig. 9C, E). In contrast, in 10 neurons recorded from 4 control-mCherry animals with CNO application, the average frequency and amplitude of sIPSCs was 36.84 Hz and 4.59 pA, respectively (Fig. 9C, E). The results showed that neither the average frequency nor the amplitude of sIPSCs was altered with or without CNO perfusion in the Control-mCherry group (*P* > 0.05). We also observed sIPSCs in Control-hM4D(Gi) group with or without CNO application. The average frequency and amplitude of sIPSCs was 39.76 Hz and 4.59 pA respectively, as determined by recordings 10 neurons from 5 control-hM4D(Gi) animals without CNO application (Fig. 9H, J). In contrast, in 10 neurons recorded from 5 control-hM4D(Gi) animals with CNO applicating, the average frequency and amplitude of sIPSCs was 41.48 Hz, 3.72pA, respectively (Fig. 9HJ). Those results suggested that the average frequency of sIPSCs of control-hM4D(Gi) group with CNO applicating was decreased compared with that without CNO application(*P* < 0.05). However, the average amplitude of sIPSCs was not altered (*P* > 0.05).

## Discussion

Chronic pain and anxiety easily comorbid, and they may share the same neurotransmitters and biological pathways [29, 30]. In rodents, CFA-induced inflammatory pain, a classic chronic pain model, could persist from weeks to months, which makes it possible to research the comorbidity mechanism of chronic inflammatory pain and anxiety in animals [31, 32]. Parent and his colleagues demonstrated that CFA-induced chronic inflammatory pain leads to the development of anxiety-like behavior after 4 weeks, and anxiety-related behaviors could be observed in subjects in the EPM, OF, and SI tests but not in the L/D exploration test [22]. Our previous study also observed similar anxiety-like behaviors in animals when they performed the EZM and OF tests 28 d after CFA injection [21, 23]. In this paper, multiple behavior tests (OF, EZM, NSF and MBT) were used to comprehensively study chronic inflammatory pain and anxiety comorbidities in CFA rats. Anxiety-like behaviors were observed in rats not only in the EPM and OF tests but also in the NSF test and MBT. These results supported that CFA-induced chronic inflammatory pain could induce anxiety-like behavior in rats in the EZM test, OF test, NSF test and MBT. Then, we further studied the underlying mechanism of inflammatory pain and anxiety comorbidity.

The ACC is well known to play an important role in pain and pain-related anxiety, depending on its specific anatomical connection, which receives nociception and emotional information from the amygdala, thalamus and other cortical areas related to pain [7, 33, 34]. In imaging and neurophysiologic studies, the excitability of the ACC was enhanced when the patient or animals were in chronic pain or had mental disorders [35–38]. In this study, the number of bilateral c-Fos- and CaMKII-positive cells in the ACC was increased, and the firing rates of PNs in ACC V layer in response to prolonged depolarizing current injection of increasing amplitude were also enhanced, accompanied by anxiety-like behaviors. All these results suggested that PNs in the ACC were activated by chronic inflammatory pain induced by CFA, which is consistent with previous studies [32, 33]. Furthermore, the increased input resistance and the decreased sIPSCs frequency of PNs indicated that the enhanced excitability of PNs might be due to functional changes in GABAergic neurons because GABA can not only change conductivity but is also the main neurotransmitter of IPSCs [39].

A previous study demonstrated that the excitability of PNs in the ACC is controlled by the balance of excitatory and inhibitory inputs [40, 41]. The enhanced excitability of PNs could be due to a deficiency in GABAergic system activity in this area [28, 42]. A previous study demonstrated that inflammatory pain stimulation affected spontaneous GABAergic plasticity at presynaptic terminals in the ACC [17]. In this study, the results also indicated that presynaptic plasticity was changed by persistent peripheral inflammatory stimulation because the frequency of sIPSCs in PNs was decreased but not the amplitude (Fig. 3F, H). The capability of GABA synthesis and release principally contributes to the function of the GABAergic system [43, 44]. However, the results did not support the changes in GABA production, because a change in the expression of GAD65/67 (which decarboxylates glutamate to produce GABA) was not observed. Therefore, we hypothesize that the reduced excitability of presynaptic GABAergic neurons, which reduces the GABA release and leads to a reduction in inhibitory presynaptic transmission in the ACC, may be the initial reason for the negative emotion in CFA rats.

Before further investigation, we first tested whether and how the GABAergic system in the ACC was involved in inflammatory pain and related negative emotion. Although GABA is a well-known major inhibitory neurotransmitter in both the central and peripheral nervous systems, the contribution of GABA to pathological pain and its related negative emotion is still controversial. In the peripheral nervous system, including the peripheral and central termini, activation of the GABA system or an increase the GABA release could alleviate various types of pathological pain [45]. However, in some conditions, activation of the GABA system may enhance pain behaviors [46, 47]. In the supraspinal nervous system, a number of studies have suggested that alterations in the GABAergic system are implicated in the pathogenesis of psychiatric and chronic pain [48–51]. However, its role is still controversial. A growing amount of evidence suggests that GABAergic interneurons could alleviate pain-related emotion by decreasing the activity of PNs in these related nuclei. However, some reports have indicated that GABAergic neurons may contribute to pain-related negative emotion. For instance, lesion of GABAergic neurons in the ACC could block formalin-induced conditioned place avoidance (CPA) and contextual fear induced by conditioning with brief footshocks [52]. In this study, microinjecting muscimol into the ACC could relieve the multiple anxiety-like behaviors of CFA rats in routine assays, including the OF (that is, increased time in the central area), EZM (increased distance traveled in the open arm and time spent in the open arm), NSF (decreased latency to feed) and MBT (increased number of buried marbles), just as a result of microinjection of muscimol into the amygdala [53]. This result indicated that activation of the GABAergic system in the ACC plays an important role in mitigating negative emotions. However, muscimol failed to regulate the inflammatory pain induced by CFA when it was injected into the ACC but not the amygdala [51]. Furthermore, blocking the function of GABA_A_R in the ACC by picrotoxin microinjection is enough to induce anxiety-like behavior in the noamal rats in the OF test, EZM test, NSF test and MBT. However, similar to muscimol microinjection, regulation of the function of the GABA system in the ACC by pharmacological methods failed to regulate the PWT in rats. All of the above results supported that the GABA system in the ACC was involved in the anxiety-like behavior induced by inflammatory pain but not the production of inflammatory pain. Activation of GABAergic interneurons in the ACC may block anxiety-like behavior in CFA rats.

Then, a designer receptor exclusively activated by designer drugs (DREADD) method was applied to investigate whether GABAergic neurons were involved in anxiety-like behaviors. Previous studies have demonstrated that GABAergic interneurons can modulate the excitability of pyramidal neurons [54, 55]. Other reports have shown that GABAergic interneurons act as a pivotal brake on excitatory signaling through inhibition of pyramidal neurons [56]. In this study, chemogenetic activation of GABAergic interneurons not only affected pain-induced anxiety-like behaviors in OF, EZM, NSF and MBT sessions but also significantly alleviated inflammatory pain in rats. However, chemogenetic inhibition of GABAergic neurons in the ACC only induced anxiety-like behaviors in normal rats in all the tests but failed to affect the pain threshold. These results directly indicated that GABAergic interneurons in the ACC were implicated in CFA-related anxiety-like behavior and that improving its excitability might be an important therapeutic target. Furthermore, the IF and patch clamp results supported that chemogenetics promoting the excitability of GABAergic neurons can significantly decrease the activation of ACC neurons, and the effect may be due to the recovery of the sIPSCs frequency. This is consistent with a previous study about on GABAergic neuron function in the brain [57]. In addition, inhibiting GABAergic neurons can significantly decrease the sIPSCs frequency of PNs in the ACC, which is consistent with the changes induced by the chronic inflammatory pain [17], and supportes the previous results that persistent inflammatory pain affects GABAergic neurons. Furthermore, inhibiting GABAergic neurons significantly activated ACC neurons, which suggested that the reduced excitability of presynaptic GABAergic neurons was able to produce anxiety-like behaviors in rats in all the tests. All these results directly indicated that the GABAergic system in the ACC was implicated in the CFA related anxiety-like behavior.

The above results hint an interesting phenomenon that pharmacological activation of GABA_A_R in the ACC region only relieved anxiety-like behaviors but not affect chronic inflammatory pain. However, chemogenetic activation of GABAergic neurons in the ACC alleviated chronic inflammatory pain and pain-related anxiety-like behaviors. Previous also observed similar results when testing the role of ACC played in the pain-related emotion [58, 59]. The muscimol could only activated the GABA_A_R which majority expressed in the synaptic and contributed to the function of synaptic transmission [60]. However, when the GABAergic neurons were activated by the chemogentic manipulation, amount of GABA was secreted from the neurons that may not only activated the GABA_A_R at the postsynaptic membrane, but also overflow the synaptic cleft and affect the extrasynaptic system such as the astrocyte and microglia expressing GABA_B_R, GABA neurotransmitter, GABA transporter [61–64]. In addition, astrocyte could be recruited by GABAergic neurons activities, and by releasing different gliotransmitters (glutamate, ATP, adenosine) they could participate from synaptic transmission [65]. Since the results could not exclude the role of GABA_B_R or GABA transporter played in the pain-related emotion or hyperalgesia, we will further study about it.

To unravel the link between GABA release and the excitability of ACC neurons in CFA-related anxiety rats, we observed patch-clamp recordings combined with chemogenetic technology with or without CNO. Our results showed that chemogenetic inhibition of GABAergic neurons in the ACC could increase the excitability of ACC neurons from control-hM4D(Gi)-CNO rats, such as the firing rates in response to prolonged depolarizing current injections of increasing amplitudes were significantly larger, and input resistance was increased. As expected, GABA release was decreased in the control-hM4D(Gi) group with CNO application, such as the average frequency of sIPSCs was decreased. Furthermore, chemogenetic activation of GABAergic neurons in the ACC inhibited the excitability of ACC neurons from model-hM3D(Gq)-CNO rats, and GABA release was increased. These results indicated our hypothesis that GABA release is decreased, leading to increased excitability of ACC-induced negative emotion.

## Conclusion

We demonstrate that the GABAergic system mediates a reduction in inhibitory presynaptic transmission in the ACC, which leads enhanced the excitability of pyramidal neurons in the ACC and is associated with chronic inflammatory pain-related anxiety.

## Supplementary Information

**Additional file 1: Fig.S1. **PV positive cells were decreased in the ACC with chronic inflammatory pain. (A) Representative figures of PV and c-Fos positivecells in the bilateral ACC in the Control and Model group (whole figure scalebars = 500 μm; local figure scale bars = 50 μm). (B) Quantificationof the IF results for PV-positive cells, and (C) its co-expression with c-Fospositive cells in the ipsilateral and contralateral ACC layer II/III-V. (D) Quantification of the IF results for PV-positive cells, and (E) its coexpression with c-Fos positive cells in the ipsilateraland contralateral ACC layer V. All data represent the mean ± SEM, n = 3. * *P*< 0.05, ** *P *< 0.01, compared to the Control group.

**Additional file 2: Fig. S2.** Intra-ACC injection of GABA_A_R antagonist induced anxiety-like behavior. (A) A schematic of the experimental design. (B, C) Representative figures show the anatomical localization of the ACC. (D) PWTs of normal animals that received picrotoxin (GABA_A_R antagonist) injections. (E) Quantification of behavioral parameters in the OF. (a) the percentage of distance in the central zone, (b) time in the centralzone, (c) the number of entries into the central zone, (d) and the totaldistance traveled throughout the arena of the Control+ACSF group and Control+picrotoxin group. (F) Quantification of behavioral parameters in the EZM. (a) the percentage of distance in the open arm, (b) time in the open arm, (c) the number of entries into the open arm of the Control+ACSF group andControl+picrotoxin group. (G) Quantification of behavioral parameters in theMBT. (H) Quantification of behavioral parameters in the NSF. (a) The time oflatency to feed, (b) and the food consumption. The trajectories of rats in theControl+ACSF group and Control+picrotoxin group group and model group in the OF(I) and EMZ (J). All data represent the mean ± SEM, n = 7. * *P *< 0.05, ** *P *< 0.01, compared to the Control+ACSF group.

**Additional file 3: Fig. S3.** The specificityof virus. (A) Representative images of VGAT cell (red)merged with GAD65/67 (green) in the ACC. (B) Representative images of VGAT cell(red) merged with CamKII (green) in the ACC. Bar = 50 μm

**Additional file 4: Fig. S4.** Chemogeneticinhibition of GABAergic neurons in the ACC causes anxiety-like behavior. (A) A schematic of the experimental design. (B)Microinjection of rAAV-VGAT1-hM4D (Gi)-mCherry into the bilateral ACC ofcontrol rats, and intraperitoneal injections of CNO (2 mg/kg body weight) inControl-mCherry-CNO and Control-hM4D(Gi)-CNO rats before behavioral test. (C) Arepresentative figure shows expression of mCherry signal in the ACC. (D) PWTschanges of control rats with CNO-mediated chemogenetic inhibition of GABAergicneurons in the ACC (n = 15 in the Control-mCherry-CNO;n = 16 in the Control-hM4D(Gi)-CNO). (E)Quantification of behavioral parameters in the OF (n = 11 in theControl-mCherry-CNO; n = 12 in the Control-hM4D(Gi)-CNO. (a) the percentage ofdistance in the central zone, (b) time in the central zone, (c) the number ofentries into the central zone, (d) and the total distance traveled throughoutthe arena of the Control-mCherry-CNO group and Control-hM4D(Gi)-CNO group. (F)Quantification of behavioral parameters in the EZM (n = 13 in the Control-mCherry-CNO; n = 13 in the Control-hM4D(Gi)-CNO. (a) The percentage of distance in the openarm, (b) time in the open arm, (c) the number of entries into the open arm ofthe Control-mCherry-CNO group and Control-hM4D(Gi)-CNO group. (G)Quantification of behavioral parameters in the NSF (n = 11 in theControl-mCherry-CNO; n = 12 in the Control-hM4D(Gi)-CNO. (a) The time oflatency to feed, (b) and the food consumption. The trajectories of rats in theControl-mCherry-CNO group and Control-hM4D(Gi)-CNO group in the OF (H) and EZM(I). Bar = 500 μm. All data represent the mean ±SEM, * *P *< 0.05, ** *P *< 0.01, compared to the Control-mCherry-CNO group.

## Data Availability

Please contact author for data requests.
