# Supplementary figures and images for: Anxiolytic effect of GABAergic neurons in the anterior cingulate cortex in a rat model of chronic inflammatory pain
Source: Mol Brain. 2021 Sep 10;14:139. doi: 10.1186/s13041-021-00849-9 (PMC8431944; doi:10.1186/s13041-021-00849-9)

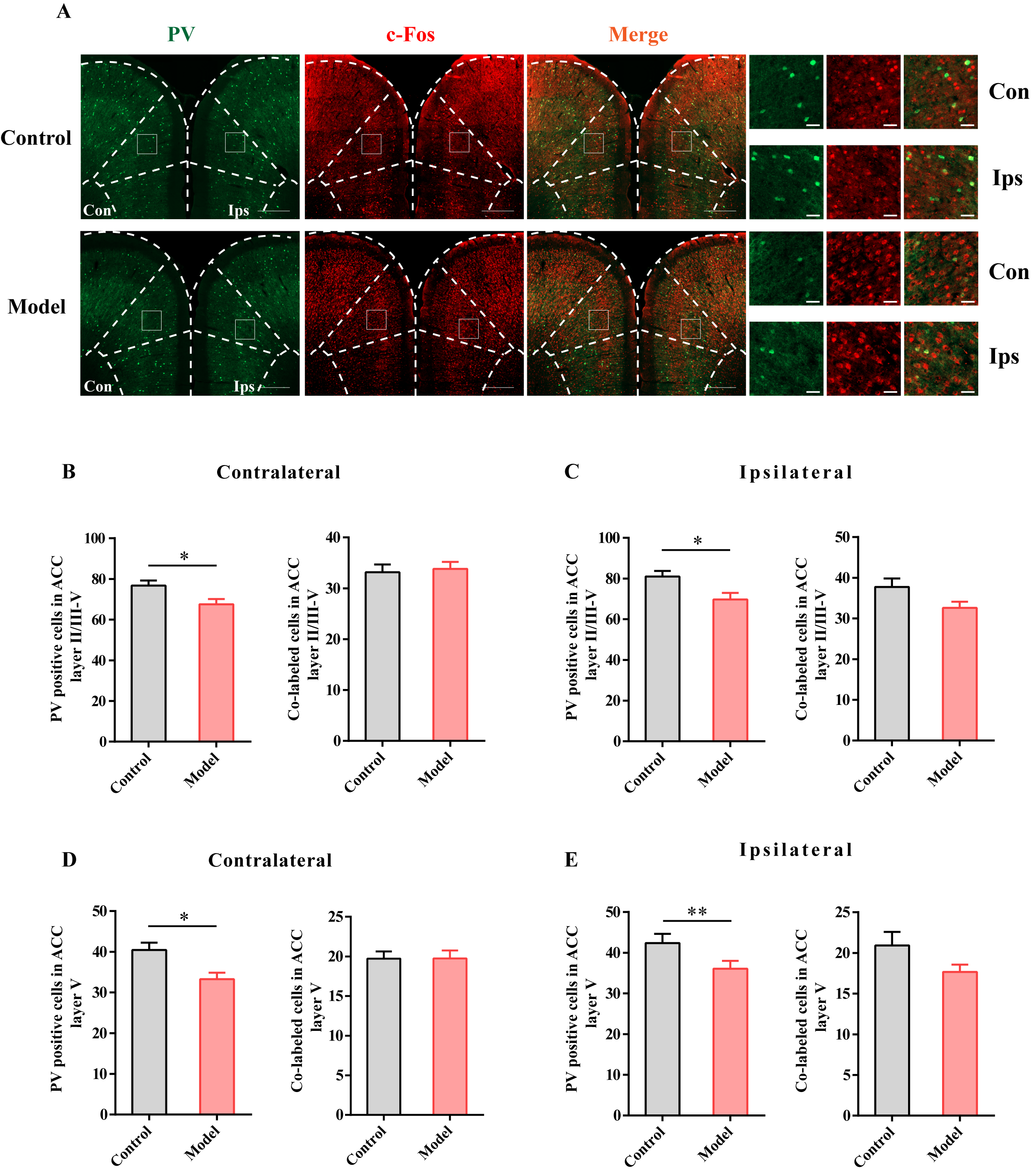

Supplement: Supplementary file 1 — Additional file 1: Fig.S1. PV positive cells were decreased in the ACC with chronic inflammatory pain. (A) Representative figures of PV and c-Fos positivecells in the bilateral ACC in the Control and Model group (whole figure scalebars = 500 μm; local figure scale bars = 50 μm). (B) Quantificationof the IF results for PV-positive cells, and (C) its co-expression with c-Fospositive cells in the ipsilateral and contralateral ACC layer II/III-V. (D) Quantification of the IF results for PV-positive cells, and (E) its coexpression with c-Fos positive cells in the ipsilateraland contralateral ACC layer V. All data represent the mean ± SEM, n = 3. * P< 0.05, ** P < 0.01, compared to the Control group. [file 13041_2021_849_MOESM1_ESM.tif]

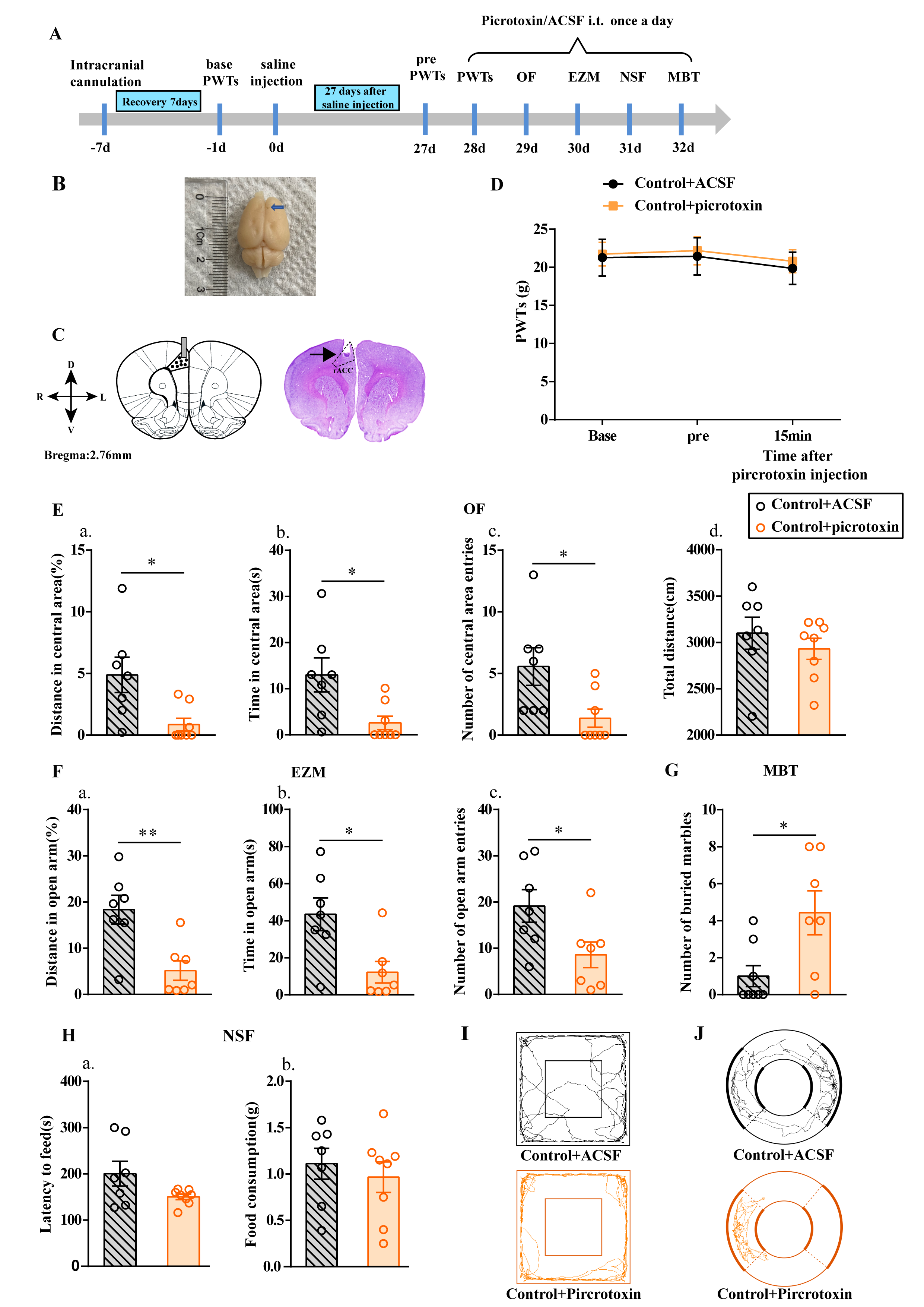

Supplement: Supplementary file 2 — Additional file 2: Fig. S2. Intra-ACC injection of GABAAR antagonist induced anxiety-like behavior. (A) A schematic of the experimental design. (B, C) Representative figures show the anatomical localization of the ACC. (D) PWTs of normal animals that received picrotoxin (GABAAR antagonist) injections. (E) Quantification of behavioral parameters in the OF. (a) the percentage of distance in the central zone, (b) time in the centralzone, (c) the number of entries into the central zone, (d) and the totaldistance traveled throughout the arena of the Control+ACSF group and Control+picrotoxin group. (F) Quantification of behavioral parameters in the EZM. (a) the percentage of distance in the open arm, (b) time in the open arm, (c) the number of entries into the open arm of the Control+ACSF group andControl+picrotoxin group. (G) Quantification of behavioral parameters in theMBT. (H) Quantification of behavioral parameters in the NSF. (a) The time oflatency to feed, (b) and the food consumption. The trajectories of rats in theControl+ACSF group and Control+picrotoxin group group and model group in the OF(I) and EMZ (J). All data represent the mean ± SEM, n = 7. * P < 0.05, ** P < 0.01, compared to the Control+ACSF group. [file 13041_2021_849_MOESM2_ESM.tif]

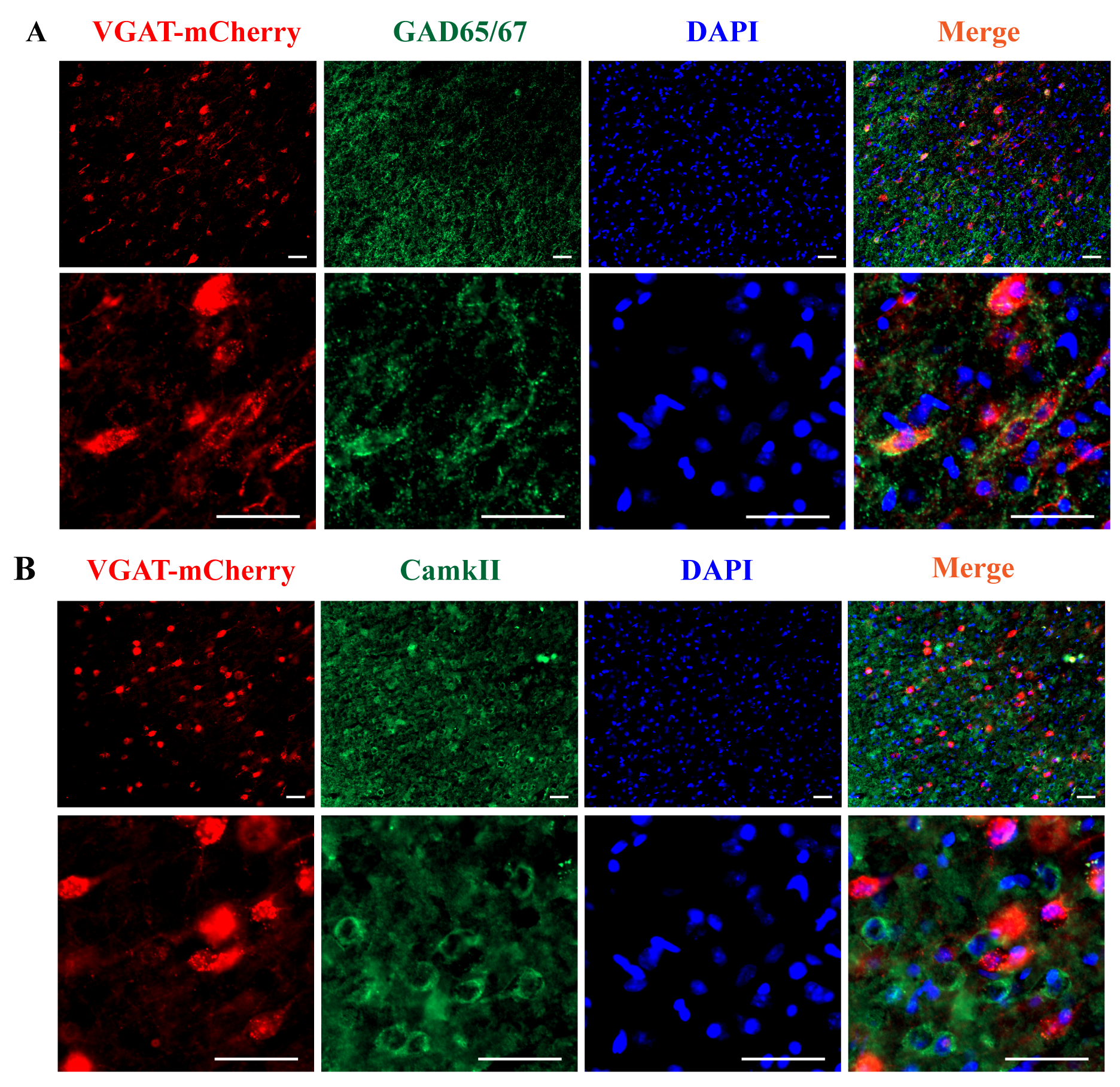

Supplement: Supplementary file 3 — Additional file 3: Fig. S3. The specificityof virus. (A) Representative images of VGAT cell (red)merged with GAD65/67 (green) in the ACC. (B) Representative images of VGAT cell(red) merged with CamKII (green) in the ACC. Bar = 50 μm [file 13041_2021_849_MOESM3_ESM.tif]

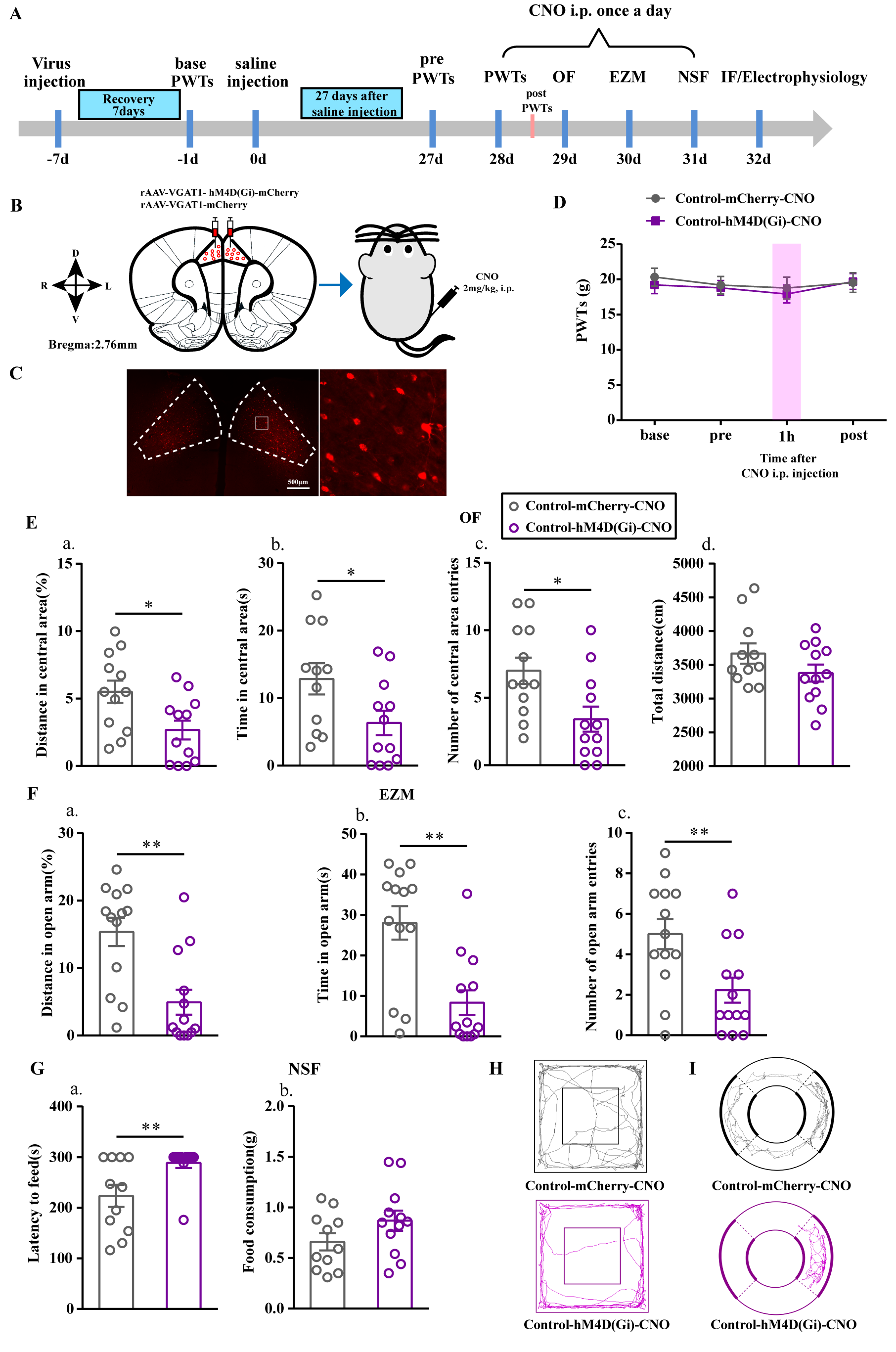

Supplement: Supplementary file 4 — Additional file 4: Fig. S4. Chemogeneticinhibition of GABAergic neurons in the ACC causes anxiety-like behavior. (A) A schematic of the experimental design. (B)Microinjection of rAAV-VGAT1-hM4D (Gi)-mCherry into the bilateral ACC ofcontrol rats, and intraperitoneal injections of CNO (2 mg/kg body weight) inControl-mCherry-CNO and Control-hM4D(Gi)-CNO rats before behavioral test. (C) Arepresentative figure shows expression of mCherry signal in the ACC. (D) PWTschanges of control rats with CNO-mediated chemogenetic inhibition of GABAergicneurons in the ACC (n = 15 in the Control-mCherry-CNO;n = 16 in the Control-hM4D(Gi)-CNO). (E)Quantification of behavioral parameters in the OF (n = 11 in theControl-mCherry-CNO; n = 12 in the Control-hM4D(Gi)-CNO. (a) the percentage ofdistance in the central zone, (b) time in the central zone, (c) the number ofentries into the central zone, (d) and the total distance traveled throughoutthe arena of the Control-mCherry-CNO group and Control-hM4D(Gi)-CNO group. (F)Quantification of behavioral parameters in the EZM (n = 13 in the Control-mCherry-CNO; n = 13 in the Control-hM4D(Gi)-CNO. (a) The percentage of distance in the openarm, (b) time in the open arm, (c) the number of entries into the open arm ofthe Control-mCherry-CNO group and Control-hM4D(Gi)-CNO group. (G)Quantification of behavioral parameters in the NSF (n = 11 in theControl-mCherry-CNO; n = 12 in the Control-hM4D(Gi)-CNO. (a) The time oflatency to feed, (b) and the food consumption. The trajectories of rats in theControl-mCherry-CNO group and Control-hM4D(Gi)-CNO group in the OF (H) and EZM(I). Bar = 500 μm. All data represent the mean ±SEM, * P < 0.05, ** P < 0.01, compared to the Control-mCherry-CNO group. [file 13041_2021_849_MOESM4_ESM.tif]
